# Chromatin landscape alteration uncovers multiple transcriptional circuits during memory CD8^+^ T-cell differentiation

**DOI:** 10.1093/procel/pwaf003

**Published:** 2025-01-13

**Authors:** Qiao Liu, Wei Dong, Rong Liu, Luming Xu, Ling Ran, Ziying Xie, Shun Lei, Xingxing Su, Zhengliang Yue, Dan Xiong, Lisha Wang, Shuqiong Wen, Yan Zhang, Jianjun Hu, Chenxi Qin, Yongchang Chen, Bo Zhu, Xiangyu Chen, Xia Wu, Lifan Xu, Qizhao Huang, Yingjiao Cao, Lilin Ye, Zhonghui Tang

**Affiliations:** Department of Hematology and Oncology, Children’s Hospital of Chongqing Medical University, National Clinical Research Center for Child Health and Disorders, Ministry of Education Key Laboratory of Child Development and Disorders, Chongqing Key Laboratory of Child Rare Diseases in Infection and Immunity, Chongqing 400014, China; Institute of Immunology, Third Military Medical University, Chongqing 400038, China; Zhongshan School of Medicine, Sun Yat-sen University, Guangzhou 510080, China; Guangzhou Women and Children’s Medical Center, Guangzhou Medical University, Guangzhou 510623, China; Zhongshan School of Medicine, Sun Yat-sen University, Guangzhou 510080, China; Institute of Precision Medicine, the First Affiliated Hospital, Sun Yat-sen University, Guangzhou 510275, China; Guangdong Province Key Laboratory of Immune Regulation and Immunotherapy, School of Laboratory Medicine and Biotechnology, Southern Medical University, Guangzhou 510515, China; Institute of Immunology, Third Military Medical University, Chongqing 400038, China; Zhongshan School of Medicine, Sun Yat-sen University, Guangzhou 510080, China; Institute of Immunology, Third Military Medical University, Chongqing 400038, China; Institute of Immunology, Third Military Medical University, Chongqing 400038, China; Institute of Immunology, Third Military Medical University, Chongqing 400038, China; Zhongshan School of Medicine, Sun Yat-sen University, Guangzhou 510080, China; Institute of Immunology, Third Military Medical University, Chongqing 400038, China; Stomatological Hospital of Chongqing Medical University, Chongqing 400015, China; Institute of Immunological Innovation and Translation, Chongqing Medical University, Chongqing 400016, China; Department of Oncology, Southwest Hospital, Third Military Medical University, Chongqing 400038, China; Institute of Immunology, Third Military Medical University, Chongqing 400038, China; Institute of Cancer, Xinqiao Hospital, Third Military Medical University, Chongqing 214426, China; Institute of Cancer, Xinqiao Hospital, Third Military Medical University, Chongqing 214426, China; Institute of Immunological Innovation and Translation, Chongqing Medical University, Chongqing 400016, China; Zhongshan School of Medicine, Sun Yat-sen University, Guangzhou 510080, China; Institute of Immunology, Third Military Medical University, Chongqing 400038, China; Institute of Immunological Innovation and Translation, Chongqing Medical University, Chongqing 400016, China; Guangdong Province Key Laboratory of Immune Regulation and Immunotherapy, School of Laboratory Medicine and Biotechnology, Southern Medical University, Guangzhou 510515, China; Institute of Immunology, Third Military Medical University, Chongqing 400038, China; Zhongshan School of Medicine, Sun Yat-sen University, Guangzhou 510080, China

**Keywords:** T cell, memory, epigenome, chromatin, interactions

## Abstract

Extensive epigenetic reprogramming involves in memory CD8^+^ T-cell differentiation. The elaborate epigenetic rewiring underlying the heterogeneous functional states of CD8^+^ T cells remains hidden. Here, we profile single-cell chromatin accessibility and map enhancer-promoter interactomes to characterize the differentiation trajectory of memory CD8^+^ T cells. We reveal that under distinct epigenetic regulations, the early activated CD8^+^ T cells divergently originated for short-lived effector and memory precursor effector cells. We also uncover a defined epigenetic rewiring leading to the conversion from effector memory to central memory cells during memory formation. Additionally, we illustrate chromatin regulatory mechanisms underlying long-lasting versus transient transcription regulation during memory differentiation. Finally, we confirm the essential roles of *Sox4* and *Nrf2* in developing memory precursor effector and effector memory cells, respectively, and validate cell state-specific enhancers in regulating *Il7r* using CRISPR-Cas9. Our data pave the way for understanding the mechanism underlying epigenetic memory formation in CD8^+^ T-cell differentiation.

## Introduction

Upon acute infection or effective vaccination, naïve antigen-specific CD8^+^ T cells engage in a dramatic and tightly orchestrated activation process, triggering a short-term effector response, and ensuing long-term immunological memory (Kaech and Cui, 2012). When naïve antigen-specific CD8^+^ T cells encounter cognate antigens, they undergo explosive clonal expansions, acquire effector functions, and differentiate into heterogeneous cytotoxic effector T cells. Following antigen clearance, the pool of activated effector T cells contracts, and a small population of long-lasting memory T cells survives that loses many characteristics associated with activated effector cells but maintains a subset of features enhancing responsiveness and protection from reinfection with the same antigen (Joshi et al., 2007). How epigenetic mechanisms acting in concert with transcription factors (TFs) to determine the developmental trajectory and define the cell state-specific functional properties of CD8^+^ T cells as they differentiate from naïve to effector and memory states are long-standing central questions.

Acute-resolving infection models have provided insights into naïve T-cell activation and differentiation into effector and long-lasting memory T cells. Reverse genetic studies in those models have identified numerous TFs involved in regulating CD8^+^ T-cell fate diversification, including *Tbx21*, *Bimp1*, Id*2*, *Stat4*, and *Zeb2* in effector cells (Intlekofer et al., 2005; Joshi et al., 2007; Mollo et al., 2014; Omilusik et al., 2015; Rutishauser et al., 2009; Y. Chen et al., 2018; Yang et al., 2011) and *Tcf7*, *Fox1*, *Zeb1*, *Bach2*, *Eomes*, *Bcl6*, *Stat3*, and Id*3* in memory cells (Cui et al., 2011; Feng et al., 2011; Guan et al., 2018; Hess Michelini et al., 2013; Ichii et al., 2002; Roychoudhuri et al., 2016; Zhou et al., 2010). However, the roles of those TFs in the dichotomy between stable and reversible gene regulation remain largely unknown. By leveraging genome-wide profiling of chromatin states, the distinguishing features of stable and reversible modes of gene regulation were traced to cell state-specific enhancers, driving the expression of genes that contributed to cell state-specific phenotypic and functional properties (Zhang et al., 2021; Kieffer-Kwon et al., 2013; Riegel et al., 2023). Enhancers connect to their target genes through long-range chromatin interactions, orchestrating complex transcription programs (Zhang et al., 2013). For instance, the ablation of a distal enhancer upstream of *Pdcd1* essentially decreased PD1 expression in activation-induced antigen-specific CD8^+^ T cells, influencing CD8^+^ T-cell differentiation (Bally et al., 2017). These studies highlighted cell state enhancers and chromatin architecture as the essential epigenetic mechanism that regulates CD8^+^ T-cell differentiation and function, allowing for transient and stable transcriptional changes. However, several obstacles have prevented the detailed examination of the role of epigenetic regulation in CD8^+^ T-cell differentiation.

First, although the landscape of *cis*-regulatory elements has been characterized for immunophenotypically defined subsets in CD8^+^ T-cell differentiation, the data on chromatin dynamics were exclusively derived from bulk profiling (Araki et al., 2009; Russ et al., 2014; Scott-Browne et al., 2016), which masks the potential heterogeneity among individual cells. Single-cell chromatin accessibility profiling has demonstrated that variations in *cis*- and transcription factor-mediated regulation elements are associated with the regulation of gene expression in individual cells (Giles et al., 2022; Lareau et al., 2019; Satpathy et al., 2019), making it possible to uncover *cis*- and transcription factor-mediated regulation mechanisms underlying epigenetic heterogeneity in the cell fate decision during memory CD8^+^ T-cell differentiation. Second, chromatin profiling studies usually yield thousands of putative enhancers; however, there is a dramatic imbalance between the pure mapping of enhancers and our understanding of which target genes are affected and how this targeting impacts cell state-specific function. Recent studies have demonstrated that enhancers act over long distances to concatenate promoters, tethering TFs, RNAPII, and transcription cofactors to regulate transcription activity (Tang et al., 2015). Thus, genome-wide mapping of enhancer-promoter chromatin interactions is essential for pinpointing epigenetic regulatory cues in functionally distinct subsets of CD8^+^ T cells. In addition, epigenetic landscape changes in the linear dimension might alter chromatin architecture in three dimensions, which is critical for better understanding the stable and reversible modes of gene regulation during memory CD8^+^ T-cell differentiation. A comprehensive and high-resolution enhancer–promoter chromatin interaction map of distinct subsets of CD8^+^ T cells is lacking. Third, there is an urgent demand to prioritize enhancers and their corresponding genes in the regulation of distinct functional CD8^+^ T-cell states. Thus, the integration of single-cell chromatin accessibility profiling and high-resolution enhancer-promoter mapping might facilitate the prioritization of cell-state-specific enhancers, their target genes, and acting TFs in a combinatorial manner. To date, no study has investigated the single-cell chromatin accessibility landscape coupled with mapping chromatin interactions in distinct CD8^+^ T-cell types.

Here, by applying single-cell ATAC sequencing (scATAC-seq) and chromatin interaction analysis with paired-end tag (ChIA-PET) sequencing approaches, we longitudinally analyzed the chromatin landscapes and chromatin architectures of naïve, effector, and memory CD8^+^ T cells induced in the lymphocytic choriomeningitis virus (LCMV) and *Listeria monocytogenes* expressing OVA (Lm-OVA) model of acute bacterial infection. These data revealed the primary layer of epigenetic changes during memory CD8^+^ T-cell differentiation, including dynamic changes in chromatin accessibility and distinct alterations in functional promoter-enhancer interactions. Our findings indicate heterogeneity within early activated CD8^+^ T cells at the epigenetic level, with short-lived effector cells (SLECs) and memory precursor effector cells (MPECs) originating under distinct epigenetic regulations. Our study also reveals that a defined epigenetic rewiring leads to the conversion of effector memory (TEM) to central memory (TCM) during memory formation. These results demonstrated that memory cells are the progeny of effector cells during memory CD8^+^ T-cell differentiation. We prioritized the critical enhancers, transcription factors, and their target genes involved in the differentiation process by integrating scATAC-seq and ChIA-PET data. Next, we confirmed the essential roles of Sox4 and Nrf2 in developing MPEC and TEM subsets, respectively, and validated cell state-specific enhancers in the regulation of *Il7r*. Overall, this study provides new insights into the underlying epigenetic mechanism regulating memory CD8^+^ T-cell differentiation during acute viral infection.

## Results

### Early and consecutive epigenetic changes in memory CD8^+^ T-cell differentiation

**Figure 1.**
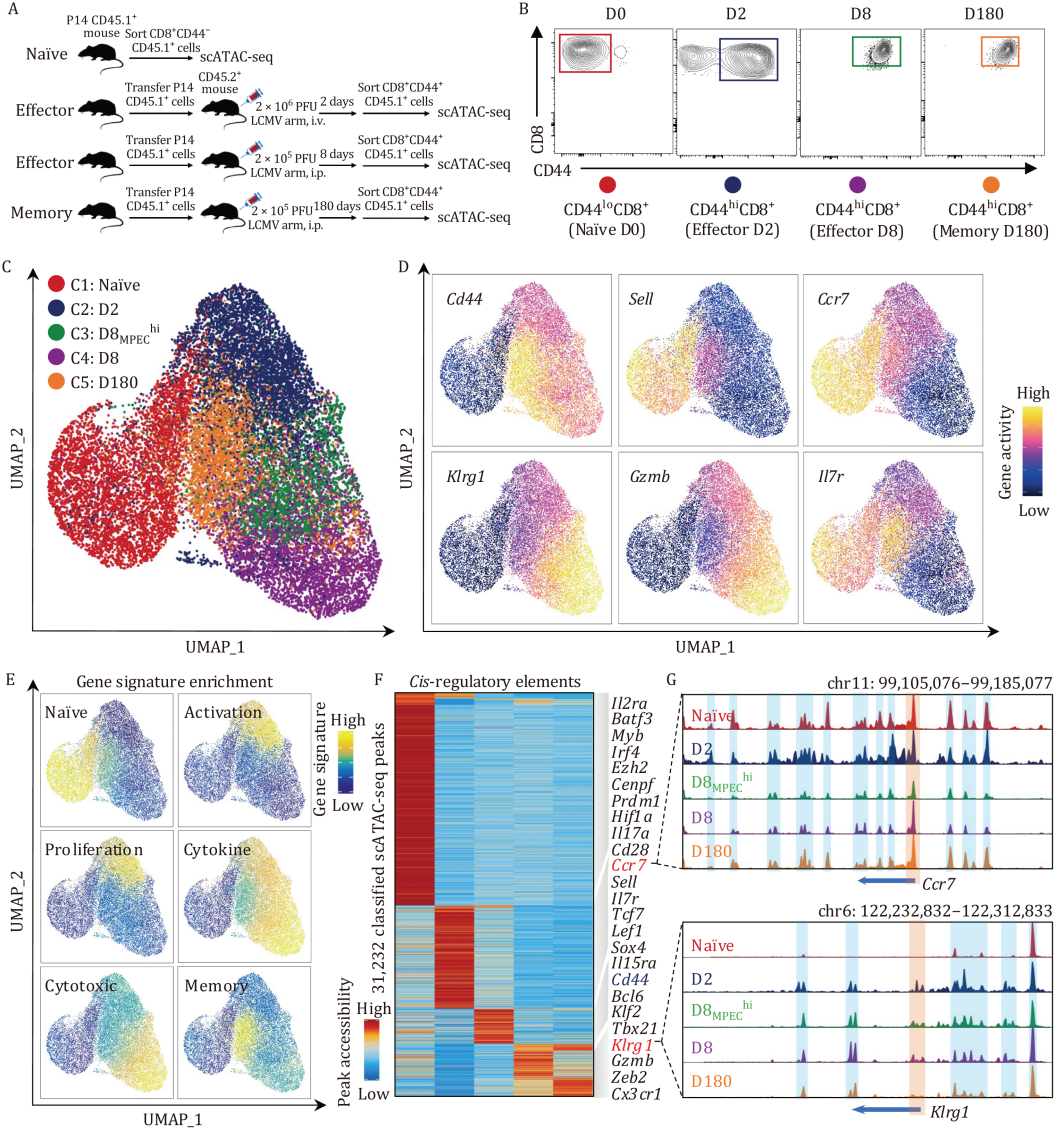
Cell state-specific epigenetic features of antigen-specific CD8^+^ T cells in response to acute viral infection. (A) Schematic overview of the experimental setup and sample collections for single-cell ATAC sequencing. Congenic distinct naïve CD45.1^+^CD8^+^ P14 cells were adoptively transferred into CD45.2^+^ hosts 1 day before LCMV-Arm^+^ infection. Splenocytes were harvested at 2-, 8-, and 180-days postinfection. Naïve T cells (CD44^lo^CD62L^hi^) were harvested from the spleen of uninfected P14 TCR transgenic mice. Antigen-experienced P14 CD8^+^ T cells (CD45.1^+^CD44^hi^) at 2-, 8-, and 180-days were sorted and applied to the 10x Genomics platform for single-cell ATAC sequencing. (B) Gating strategy for CD8^+^ T cells sorting from the spleen, cells were gated on live CD45.1^+^ cells. (C) UMAP projection of 19,950 CD8^+^ T cells from five samples with single-cell chromatin accessibility profiles. Each dot represents an individual cell, colored according to the sample of origin. (D) UMAP visualization illustrates activity scores of representative genes for CD8^+^ T-cell differentiation. Dot colors based on the log-transformed normalized gene activity scores. (E) UMAP visualization illustrates the enrichment scores of gene signatures associated with distinct CD8^+^ T cell states using scATAC-seq data. (F) Heatmap represents the chromatin-accessible peaks uniquely activated in individual samples. (G) Genome track view visualizes the chromatin accessibility at *Ccr7* and *Klrg1* loci by aggregating scATAC-seq signals from each sample. Promoters and proximal *cis*-regulatory elements are highlighted in boxes as indicated, respectively.

To investigate the dynamic epigenetic alterations of CD8^+^ T cells throughout acute viral infection, we purified naïve CD8^+^ T cells from P14 (CD45.1^+^ LCMV GP33–41, H-2D^b^ specific) transgenic mice and adoptively transferred them into wild-type C57BL/6 recipient mice (CD45.2^+^). These mice were then infected 1 day later with the lymphocytic choriomeningitis virus Armstrong (LCMV-Arm^+^) strain. P14 cells from the spleen were sorted and subjected to scATAC sequencing at 0, 2, 8, and 180 days postinfection (Fig. 1A and 1B). As MPECs generally account for around 5% of D8 CD8^+^ T cells (Kaech and Wherry, 2007), to dissect their characteristics more precisely, 20% of enriched MPECs were pooled into the D8 effector cells, referred to as the D8_MPEC_^hi^ sample. The D8 effector cells conventionally collected without adding extra MPECs were denoted as the D8 sample. Quality evaluation of scATAC sequencing libraries showed that these datasets were high quality and integrity (Fig. S1A–C). By applying stringent quality control and cell filtering, 4,693 naïve cells, 5,286 D2 cells, 2,315 D8_MPCE_^hi^ cells, 4,096 D8 cells, and 3,560 D180 memory cells were obtained for downstream analysis (Fig. S1D). After batch effect correction, multiple sample integration, and uniform manifold approximation and projection (UMAP) embedding, we acquired the overview single-cell chromatin accessibility landscapes of 19, 950 CD8^+^ T cells from five samples representing distinct CD8^+^ T-cell states (Fig. 1C). In the UMAP embedding space, naïve T cells were most distant from other T-cell populations, consistent with the fact that naïve T cells have not been activated. The D8-cell population was positioned far from naïve T cells with a distinct separation (Fig. 1C), indicating the most striking chromatin differences between naïve and fully matured effector T cells. While D8_MPEC_^hi^ and D8 cells share a largely overlapping chromatin landscape due to their overall similarity as effector T-cell populations, the enrichment of MPECs in D8_MPEC_^hi^ contributes to its distinct position in UMAP embedding space. In comparison, D180 memory T cells were distributed between the naïve, D8_MPEC_^hi^, and D8 cells in the UMAP embedding space, indicating that memory T cells are in a hybrid state at the epigenetic level.

We obtained five clusters in the UMAP embedding space using an unsupervised clustering approach (Fig. 1C). To comprehensively investigate whether these clusters defined by scATAC-seq data reflect the transcriptional gene signatures, we first performed gene activity analysis, computed from the aggregate accessibility of several enhancers linked to the target gene. Furthermore, we conducted gene signature enrichment analysis using publish available gene signatures from distinct CD8^+^ T-cell subsets (Scott-Browne et al., 2016; Szabo et al., 2019; Wolski et al., 2017). Our results showed that each stage-specific cell signature was explicitly enriched in each cluster (Figs. 1D, 1E, S1E and S1F; Table S1). These findings further confirmed that clusters defined by scATAC-seq data possessed the transcriptional gene signatures of distinct CD8^+^ T-cell subsets.

Next, to systematically investigate dynamic chromatin accessibility changes in CD8^+^ T cells during infection, we identified 105,328 unique peaks across all five samples (Fig. S1G; Table S2) and 31,232 variably accessible chromatin peaks within individual samples (Fig. 1F; Table S3). We found that D2 cells possessed the most uniquely accessible chromatin peaks (Fig. 1F), indicating widespread chromatin reprogramming during CD8^+^ T-cell activation and differentiation. Peak annotation showed that most differentially accessible regions were located in intronic and distal intergenic noncoding regions (Fig. S1H). We observed that accessible chromatin regions belonging to a particular subset were mostly linked to essential genes and transcription factors crucial for the distinct status of CD8^+^ T-cell subsets, including *Sell, Ccr7, Il7r, Tcf7,* and *Lef1* for naïve T cells*, Gzmb, Klrg1, Cx3cr1, Tbx21* and *Zeb2* for effector T cells, and *Bcl6* and *Klf2* for memory T cells (Fig. 1F and 1G).

Moreover, to further detect alterations in chromatin accessibility after CD8^+^ T-cell activation, we compared D2, D8_MPEC_^hi^, D8, and D180 cluster to the naïve T-cell cluster to detect differences in chromatin accessibility. The analysis demonstrated that memory CD8^+^ T cells (D180 cluster) had smaller changes in chromatin accessibility than activated CD8^+^ T cells (D2, D8_MPEC_^hi^, and D8 clusters) in comparison with naïve CD8^+^ T cells (Fig. S1I; Table S4), which further confirmed the above finding that compared to other CD8^+^ T-cell subsets, memory CD8^+^ T cells possessed the most naïve T-cell epigenetic characteristics after undergoing dynamic epigenetic reprogramming during differentiation. However, the gene activity of effector-associated genes such as *Klrg1*, *Gzmb*, *Prf1*, and *Tbx21* was broadly similar in D8_MPEC_^hi^, D8, and D180 memory T cells (Fig. S1D and S1E). The activity of these effector-like genes in memory T cells is likely inherited from effector cells, permitting the rapid re-expression of effector-related genes upon TCR restimulation by reinfection. We also noticed that 59,307 (56.31%) open chromatin regions were constitutive after the activation of CD8^+^ T cells, consistent with their cell identity (Fig. S1J).

In summary, we found that D2 cells displayed the most striking differences in chromatin accessibility, while memory cells possessed a hybrid epigenetic characteristic, indicating that the epigenetic reprogramming of memory cells changed strikingly from that of D2 cells but continued to advance over differentiation.

### Dynamic transcription factor-mediated regulation landscapes in memory CD8^+^ T-cell differentiation

**Figure 2.**
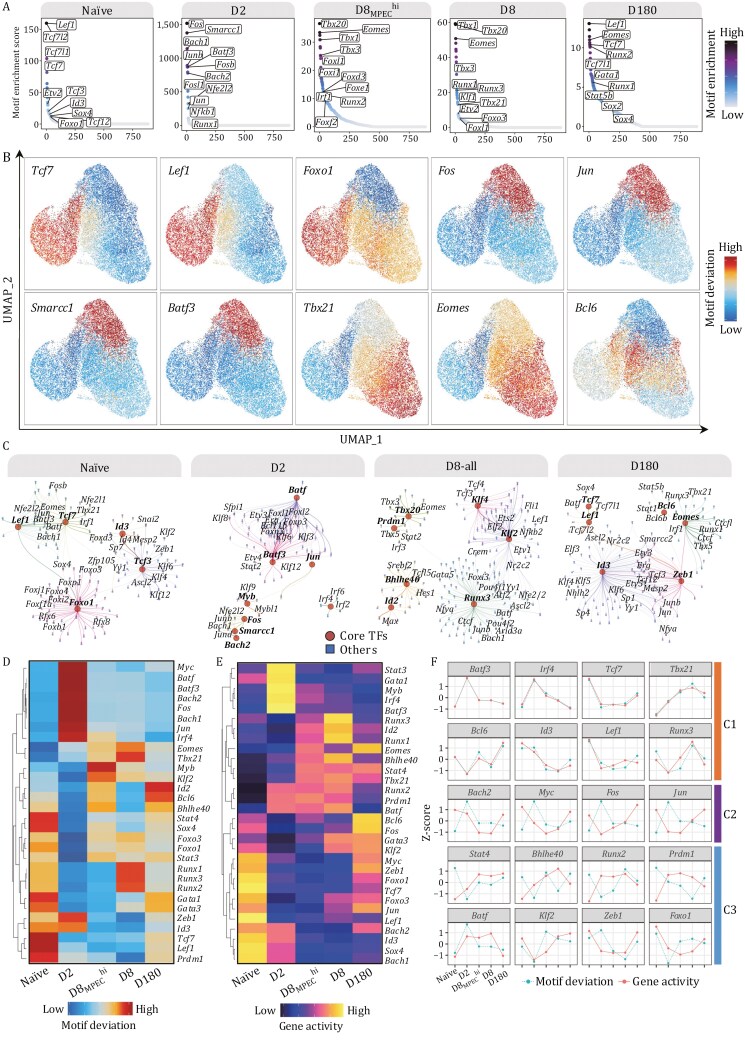
Dynamics of transcriptional factors activity in the regulation of CD8^+^ T-cell differentiation. (A) Ranking dot plot represents transcription factor (TF) motif enrichment in open chromatin regions in each sample. Rank-sorted TF motifs are shown on the x-axis, and − log_10_(FDR) of motif enrichment is shown on the y-axis. (B) UMAP projection illustrates motif deviation scores in each cell. (C) TF coexpression networks inferred by TF motif deviation in each sample. The samples D8_MPEC_^hi^ and D8 were combined as D8-all for the presentation. (D and E) Heatmap shows normalized motif deviation (D) and gene activity (E) scores of representative TFs in each sample. (F) Line chart shows normalized Z-scores of TF gene activity and the corresponding motif deviation across each sample.

We next sought to characterize the transcription factor-mediated regulation landscapes of cell state-specific TFs during CD8^+^ T-cell differentiation. First, we performed a TF motif enrichment analysis according to the unique chromatin accessibility in each cell subset. The results showed that exclusive open chromatin regions in each cell subset significantly enriched motifs for TFs playing critical roles in the corresponding cell subset. For example, the motifs of *Tcf7*, *Lef1*, and *Foxo1* were substantially enriched at accessible chromatin regions in naïve CD8^+^ T cells. In contrast, binding sites for *Fos*, *Jun*, *Smarcc1*, *Batf3* and *Nfe2l2* were highly enriched within accessible chromatin regions of D2 cells. Notably, the motifs of *Bach2* have also been enriched in D2-activated CD8^+^ T cells, reflecting that *Bach2* is recruited to enhancers after very early TCR stimulation and limits the expression of TCR-driven genes by attenuating the availability of activator protein-1 (AP-1) sites to Jun family signal-dependent TFs, restraining terminal differentiation while safeguarding memory T-cell programming (Roychoudhuri et al., 2016). Furthermore, *Tbx21*, *Eomes*, and *Runx3* binding motifs were significantly enriched in D8_MPEC_^hi^ and D8 cells. The motifs of *Tcf7* and *Lef1* were consistently enriched in D180 memory T cells, echoing their enrichment in naïve cells (Fig. 2A).

Furthermore, we calculated motif activity to predict TFs involved in gene regulation during CD8^+^ T-cell differentiation at the single-cell level using ChromVAR (Schep et al., 2017). Among the top 200 ranked TF motifs, we identified TF motifs associated with T-cell activation, differentiation, stemness, and chromatin architecture remodeling, such as *Fos*, *Junb*, *Smarcc1*, *Tcf7*, *Yy1*, and *Ctcf* (Fig. S2A; Table S5). UMAP visualization of motif activity and TF footprinting patterns further confirmed high motif enrichment of TFs that have been reported to have critical functions in each stage (Figs. 2B, S2B and S2C). This indicates that distinct T-cell states were attributed to diverse transcription factor-mediated regulation landscapes.

Although many TFs that maintain naïve CD8^+^ T-cell identity and promote differentiation have been identified, it remains unknown how those TFs regulate epigenetic reprogramming in a coordinated manner. Thus, we constructed a TF coregulatory network in each cell subset using motif deviation scores from individual cells (Fig. 2C). In naïve cells, *Tcf7* and *Lef1* were prominently enriched in the same coregulatory network and shared similar chromatin binding sites, consistent with their synergistic roles in restricting effector molecule expression and preventing aberrant cytotoxic activities in naïve CD8^+^ T cells (Xing et al., 2016). Next, after TCR engagement, *Batf* and *Batf3* both centered in the same coregulatory network in D2 cells, consistent with the finding that *Batf* and *Batf3* peaked shortly after activation and programming of effector and memory CD8^+^ T cells, respectively (Ataide et al., 2020; Tsao et al., 2022). *Runx3* was associated with multiple TFs within the coregulatory network in combined D8_MPEC_^hi^ and D8 cells; thus, *Runx3* might be critical for guarding cytotoxic CD8^+^ effector T cells against differentiation deviation toward follicular helper T cells (Shan et al., 2017). *Zeb1* and Id*3* were prominent factors with partially cooccurring TFs in the coregulatory networks of memory CD8^+^ T cells. Noticeably, *Tcf7* and *Lef1* appeared again in the coregulatory network of memory cells. However, the co-occurrence of TFs in the coregulatory network was essentially different from that in naïve T cells, indicating that although TFs exhibit the same expression profiles during cell differentiation, they may cooperate with distinct TFs to determine dynamic cell states (Fig. 2C).

To assess TF functions in regulation only permitted by the synchronization of TF activity and the accessibility of their chromatin binding sites, we then characterized the concurrence between TF activity and motif enrichment during CD8^+^ T-cell differentiation. To do so, we focused on TFs that have been demonstrated to play a critical role in CD8^+^ T-cell differentiation. TF activity and motif enrichment represented dynamic changes during differentiation (Figs. 2D–F and S2D). We categorized the targeted TFs into three distinct groups according to synchronization patterns of TF activity and binding motif enrichment. The first category, including *Bat3*, *Irf4*, *Tcf7*, *Tbx21*, *Bcl6*, and Id*3*, demonstrated a concordant pattern between TF gene activity and interacting binding motif enrichment (Fig. 2F). Interestingly, the interaction between gene activity and motif enrichment of *Tcf7* peaked in naïve and D180 cells. *Batf3* and *Irf4* peaked in D2 cells, and *Tbx21* peaked in D8 cells (Fig. 2F), indicating that TFs with the highest coordination to their interacting epigenetic elements might be most functionally important in the respective cell subsets. In the second group, TF gene activity occurred prior to the accessibility of their chromatin binding sites, such as *Bach*, *Myc*, *Fos*, and *Jun*. Primarily, TF gene activity peaked in naïve cells, while the chromatin binding sites became most accessible in the subsequent D2 cell stage. Conversely, in the third group, TF gene activity occurred after the opening of chromatin binding sites. These TFs included *Stat4*, *Bhlhe440*, *Runx2*, and *Prdm1* (Fig. 2F).

Taken together, interacting patterns between TF activities and the opening of their chromatin binding sites could be characterized into synchronized and preprogramming models. In the preprogramming model, TF activity or the opening of chromatin sites occurs first, likely permitting the immediate initiation of cell differentiation when the appropriate chromatin binding sites or TFs emerge.

### Epigenetic lineage bifurcation of effector and memory CD8^+^ T cells at the early stage

Memory T cells inherit vital features of both naïve and effector cells, such as persisting for extended periods after the antigen has been cleared, the rapid elaboration of effector function, and the ability to rapidly and extensively proliferate when re-encountering the same pathogens (Ahmed and Gray, 1996).

**Figure 3.**
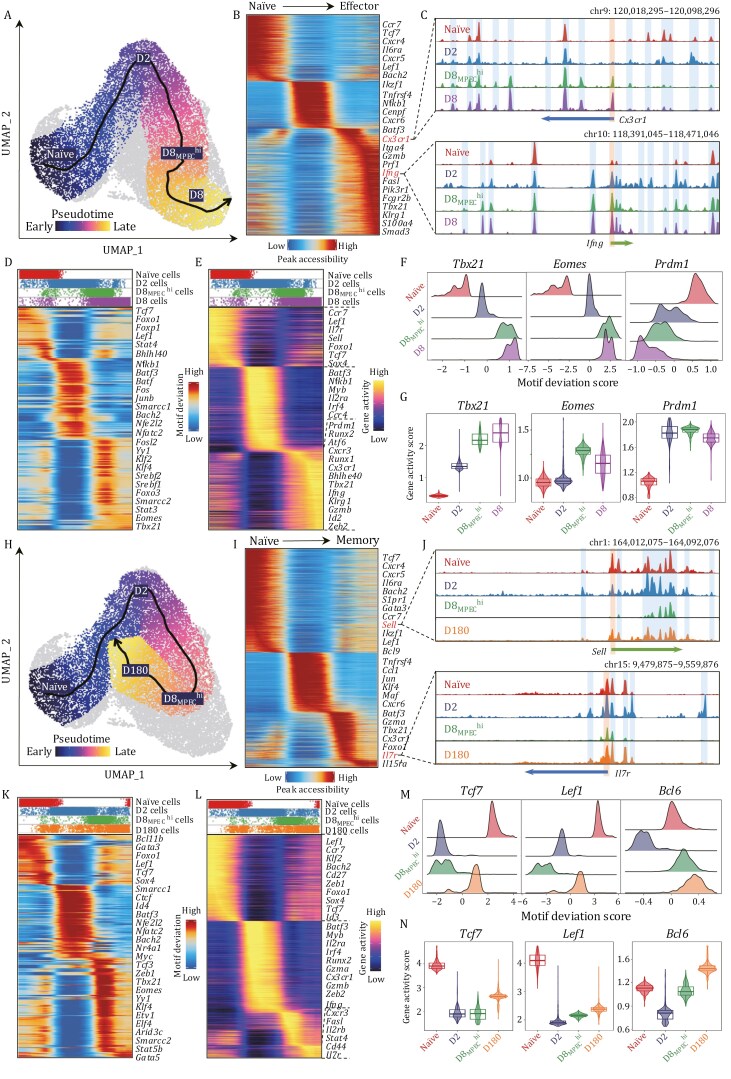
Distinct epigenetic trajectories for developing effector and memory CD8^+^ T cells. (A) UMAP visualization shows the inferred differentiation trajectory from naïve to effector CD8^+^ T cells. UMAP embedding overlaid with pseudotime values of cells. The smoothed line with the arrow represents the predicted trajectory path. (B) Heatmap displays the dynamic chromatin accessibility along the pseudotime in the differentiation trajectory from naïve to effector CD8^+^ T cells. Notable genes are indicated near their approximate position in the heatmap. (C) Genome track view visualizes the chromatin accessibility at *Cx3cr1* and *Ifng* loci by aggregating scATAC-seq signals from each sample. Promoters and *cis*-regulatory elements are highlighted in boxes as indicated. (D and E) Heatmap shows TF motif deviation (D) and gene activity (E) scores along pseudotime in the differentiation trajectory from naïve to effector CD8^+^ T cells. Notable motifs and genes are indicated near their approximate positions in the heatmaps. The top panel denotes the cell distribution along the pseudotime trajectory from each sample. (F) Ridge plot denotes the notable distribution of TF motif deviation scores across each sample. (G) Violin plot shows representative TF gene activity scores across each sample. The boxplots denote the medians and the quartile range (25%–75%), and the length of whiskers represents 1.5× the IQR. (H) UMAP visualization displays the inferred differentiation trajectory from naïve to memory CD8^+^ T cells. Similar to (A). (I) Heatmap shows the dynamic chromatin accessibility along the pseudotime in the differentiation trajectory from naïve to memory CD8^+^ T cells. Similar to (B). (J) Genome track view visualizes the chromatin accessibility at *Sell* and *Il7r* loci by aggregating scATAC-seq signals from each sample. Similar to (C). (K and L) Heatmap shows TF motif deviation (K) and gene activity (L) scores along pseudotime in the differentiation trajectory from naïve to memory CD8^+^ T cells. Similar to (D and E). (M) Ridge plot denotes the *Tcf7*, *Lef1*, and *Bcl6* motif deviation scores in each sample. (N) Violin plot shows *Tcf7*, *Lef1*, and *Bcl6* gene activity scores in each sample.

We reason that profiling chromatin accessibility might reveal hidden layers of regulation governing CD8^+^ T-cell lineage development. Therefore, we applied pseudotime approach in which time-series labels of samples were used as an input for the regression model to reconstruct CD8^+^ T-cell differentiation trajectories. The results revealed a pseudotime trajectory originating from naïve T cells and bifurcating into two branches: (i) terminal effector T-cell fates and (ii) memory T-cell fates, which were denoted as effector and memory trajectories, respectively (Figs. 3A, 3H and S3A). Through comparison, we revealed both shared and unique chromatin accessibility patterns and TF activity reprogramming across those two diverged trajectories (Fig. 3B–G and 3I–N). For example, *cis*-elements with early accessibility in the effector and memory trajectories included *Foxo1*, *Tcf7*, *Il7r*, and *Sell*, critical factors for homeostasis or circulation in naïve cells (Fig. 3B and 3I). In addition, *cis*-elements associated with critical TFs for effector cell fate determination (*Tbx21, Prdm1,* Id*2,* and *Zeb2*) and characteristic features (*Cx3cr1* and *Ifng*) were uniquely accessible in the late effector trajectory (Fig. 3B–G).Memory T-cell commitment was accompanied by the additional accessibility of *Sell* and *Il7r* and motif enrichment of *Tcf7* and *Lef1* (Fig. 3I–N), which are important for memory T-cell differentiation. While *Bcl6* also displayed a distinct motif enrichment pattern, its role appears more prominent in the D8_MPEC_^hi^ population compared to *Tcf7* and *Lef1*, this is consistent with recent finding that *Bcl6* is required for the generation of CD8^+^ memory precursors upon acute viral infection (Liu et al., 2019). Based on the synchronized patterns between TF activity and accessibility of their targeted chromatin binding sites in the trajectory, we determined multiple TFs that were essential in epigenetic regulation for each CD8^+^ T-cell stage. For example, *Tcf7* and *Lef1* were identified in naïve T cells, consistent with their roles in T-cell development and quiescence (Feng et al., 2011), followed by *Tbx21* and *Yy1* in effector T cells, recapitulating their known functions in effector T-cell activation and differentiation (Fig. S3B). In contrast, *Foxo1, Tcf7,* and *Lef1* were again observed in memory cells, mostly mirroring their appearance in naïve cells (Fig. S3C).

Next, to validate how applicable the current data might be to other infection, we purified naïve CD8^+^ T cells from OT-1 (CD45.1^+^ OVA257-264, H-2Kb specific) transgenic mice and adoptively transferred them into wild-type C57BL/6 recipient mice (CD45.2^+^). These mice were then infected 1 day later with Lm-OVA. OT-1 cells from the spleen were sorted and subjected to scATAC sequencing at Day 0, 2, and 8 postinfection. After applying stringent quality control, cell filtering, and batch effect correction, 35,177 high-quality OT-1 CD8^+^ T cells from three samples (11,165 naïve cells, 11,594 D2 cells, and 12,418 D8 cells) were obtained for downstream analysis. PCA analysis showed these CD8^+^ T cells clustered separately by their time point, irrespective of whether it is originated from P14 or OT-1 cells, which demonstrated the highly similar characteristics of CD8^+^ T cells in two different kinds of infections (Fig. S4A). UMAP visualization showed naïve T cells discrete from D2 and D8 cells, this is in accordance with the widespread chromatin accessibility changes during activation as seen in the P14 model (Fig. S4B). According to the promoter’s chromatin accessibility of *Klrg1* and *Il7r*, D8 OT-1 cells were divided into MPECs and SLECs (Fig. S4B and S4C). Next, we applied a supervised pseudotime approach to reconstruct CD8^+^ T-cell differentiation trajectories in OT-1 infection model. The results revealed a pseudotime trajectory originating from naïve T cells and bifurcating into two branches: (i) SLEC and (ii) MPEC fates, which were denoted as SLEC and MPEC trajectories, respectively (Fig. S4D and S4H). In regards to the TFs that are important for MPEC formation, such as *Tcf7, Sox4,* Id*3*, and *Zeb1*, we observed a steady gene activity decrease from naïve to D2 and a smooth increase from D2 to MPECs (Fig. S4E–G). For those TFs that are vital for SLEC trajectories, we revealed a steady increase in gene activity of *Tbx21, Runx1, Bhlhe40, and Zeb2* which demonstrated our analysis can indeed reflect the biological functions (Fig. S4I–K).

Overall, our findings reveal that the early activated CD8^+^ T-cell population includes MPECs, initiating a divergent trajectory to memory T-cell development distinct from the well-recognized process effector cell development (Fig. S3D).

### Early effector CD8^+^ T cells produce MPECs and SLECs

Previous studies have demonstrated that the expression of the cytokine receptors IL-7R and KLRG1 can distinguish two effector T-cell subsets with distinct memory potentials, SLECs (KLRG1^hi^IL-7R^lo^) and MPECs (KLRG1^lo^IL-7R^hi^) (Joshi et al., 2007), concordant with the bifurcated trajectories established above. However, how epigenetic regulation precisely controls the divergent origins of these two subsets (SLECs and MPECs) of early activated CD8^+^ T cells remains largely unknown.

**Figure 4.**
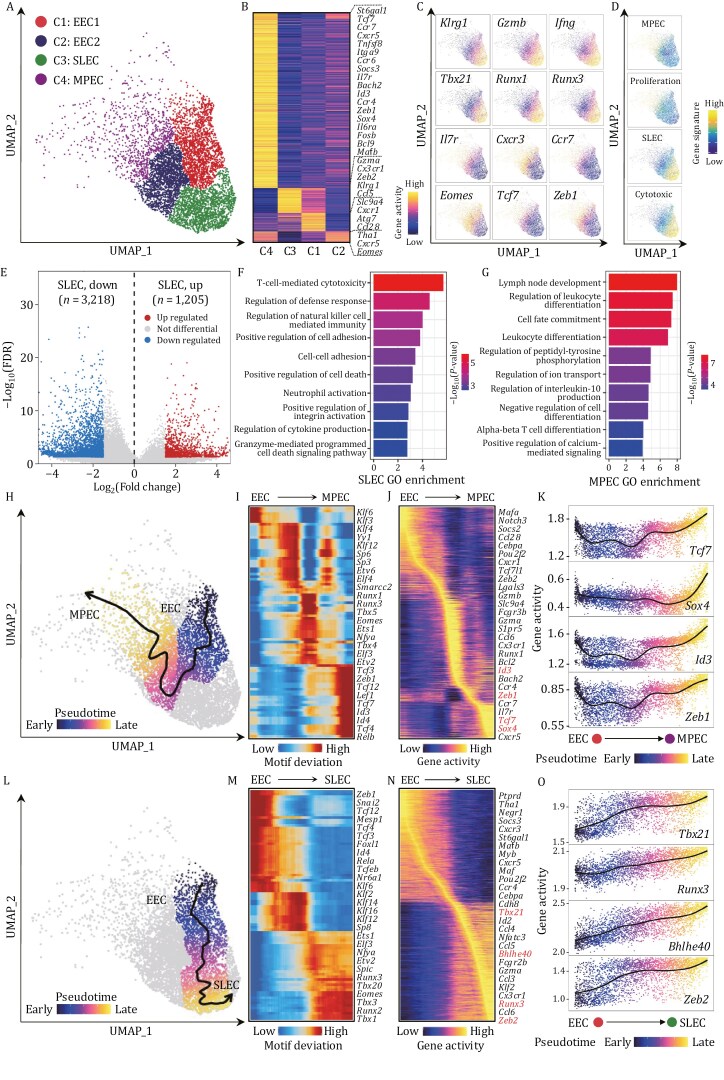
Effector CD8^+^ T cells exhibit epigenetic heterogeneity during differentiation. (A) UMAP projection shows clustered effector CD8^+^ T cells from D8_MPEC_^hi^ and D8 samples. Dots represent individual cells and colors indicate cluster identity. EEC1 and EEC2: *Klrg*^lo^*Il7r*^lo^; SLEC: *Klrg1*^hi^*Il7r*^lo^; MPEC: *Klrg1*^*l*o^*Il7r*^hi^. (B) Heatmap shows normalized activity scores of genes that exhibited distinct chromatin accessible in each cluster from (A). Notable genes for each cluster are indicated near their approximate position in the heatmap. (C) UMAP visualization illustrates activity scores of representative genes for each cluster in (A). The gene activity score was calculated by the aggregate accessibility of peaks linked to the associated genes, indicating the gene transcription activities. Dot colors based on the log-transformed normalized gene activity scores. (D) UMAP visualization displays enrichment scores of gene signatures for each cluster in (A). (E) Volcano plot shows the differential chromatin accessible peaks between SLEC (C3 in A) and MPEC (C4 in A) cells. (F and G) Bar plot shows GO enrichments of biological processes in SLEC (F) and MPEC (G) up-regulated genes. (H) UMAP visualization displays the inferred differentiation trajectory from EEC to MPEC cells. UMAP embedding overlaid with pseudotime values of individual cells. The smoothed line with the arrow represents the predicted trajectory path. Except for EEC and MPEC cells, the other cells are colored gray in the UMAP embedding. (I and J) Heatmap shows motif deviation (I) and gene activity (J) scores along pseudotime from EEC to MPEC differentiation trajectory. (K) Scatter plot shows ordered activity scores of representative genes over pseudotime of the differentiation trajectory from EEC to MPEC cells. A smoothing line represents loess regressions for gene activity scores. Each dot represents an individual cell colored with pseudotime score. (L) UMAP visualization displays the inferred differentiation trajectory from EEC to SLEC cells. Similar to (H). (M and N) Heatmap shows motif deviation (M) and gene activity (N) scores along pseudotime from EEC to SLEC differentiation trajectory. (O) Scatter plot shows ordered activity scores of representative genes over pseudotime of the differentiation trajectory from EEC to SLEC cells. Similar to (K).

Thus, we focused on D8_MPEC_^hi^ and D8 samples, comprising fully activated CD8^+^ T cells, to distinguish between SLEC and MPEC subsets. We reclustered cells from the D8_MPEC_^hi^ and D8 samples into four clusters, annotating the clusters with well-established CD8^+^ T-cell state marker genes (Figs. 4A and S5A). We observed the highest activity of SLEC marker genes in cluster 3, including *Klrg1, Gzmb, Ifng, Tbx21, Runx1,* and *Runx3.* In contrast, MPEC marker genes, such as *Il7r, Cxcr3, Ccr7, Eomes, Tcf7,* and *Zeb1*, were primarily active in cluster 4 (Figs. 4B, 4C and S5B; Table S6). These findings imply that clusters 3 and 4 represented SLECs and MPECs, respectively. Clusters 1 and 2 displayed the intermediate signatures of both MPECs and SLECs, and we defined them as two subsets of early effector T cells (EECs) (Plumlee et al., 2015). Furthermore, we calculated gene signature scores for distinct effector T-cell states in individual cells, projecting into the UMAP embedding space for visualization. Cluster 4 showed both MPEC and proliferation signature enrichments (Fig. 4D; Table S1), in line with the cooccurring signatures previously identified in murine CD8^+^ T cells during acute infection (Scott-Browne et al., 2016; Szabo et al., 2019; Wolski et al., 2017). Cluster 3 uniformly displayed SLEC and cytotoxicity signatures (Fig. 4D). By directly comparing the differentially accessible chromatin regions, we detected 3,218 and 1,205 exclusively accessible peaks in MPECs and SLECs, respectively (Figs. 4E and S5C–E; Table S7), which were then applied to identify genes with differential activity between these subsets. Gene ontology (GO) enrichment analysis of differentially activated genes indicated that SLECs were mostly enriched in T-cell-mediated cytotoxicity and regulation of defense response. In contrast, MPECs were significantly enriched in lymph node development and regulation of leukocyte differentiation functional pathways (Fig. 4F and 4G). Noticeably, TF motifs associated with differentially activated genes were also enriched in MPECs or SLECs in a concordant manner. For instance, *Foxo1*, *Sox4*, Id*3*, *Tcf7*, and *Zeb1* binding motifs were primarily enriched in MPECs, while *Jun*, *Tbx21*, and *Runx3* binding motifs were prominently enriched in SLECs (Fig. S5F and S5G). We hypothesized that the differences in epigenetic properties between MPECs and SLECs might result in bifurcated branches for cell fate commitments, as mentioned above (Fig. S3A). To this end, we further explored the epigenetic regulatory mechanism underlying the divergent development of MPECs and SLECs in early activated CD8^+^ T cells by inferring the supervised pseudotime trajectory. Indeed, we obtained a divergent trajectory originating from the EEC1 subset that subsequently split into MPEC or SLEC cell fates (Figs. 4H, 4L and S5H). We found the distinct chromatin accessibility profiles and transcription factor activities that define the divergent pathways leading to these two subsets. Based on the trajectory toward MPEC, we identified multiple known and novel TFs involved in epigenetic regulation to dictate MPEC fate. We found that some members of the KLF family exhibited different TF motif enrichment between MPECs and EECs (Fig. 4I). Furthermore, chromatin binding sites for *Zeb1*, *Lef1*, *Tcf7*, Id*3*, and Id*4* were not present in EECs but converted to accessible sites in MPECs (Figs. 4I and S5I). In parallel, *Tcf7*, *Sox4*, Id*3*, and *Zeb1* represented a steady increase while *Zeb2* showed a steady decrease in gene activity during the developmental transition from EEC to MPEC (Fig. 4J and 4K). However, in the trajectory toward SLECs, we observed a steady increase in the gene activity of *Tbx21, Bhlhe40, Runx1, Zeb2, Runx3,* and transcription factor-mediated regulation elements associated with known TFs play a critical role in the development of cytotoxic T-cell lineages *Runx3* and novel members from the KLF family, such as *Klf6*, *Klf12*, *Klf14*, and *Klf16* (Figs. 4L–O and S5J).

Overall, our findings delineate the dynamic epigenetic regulation that controls the divergent development of SLECs and MPECs and identified potential novel TFs leading this process.

### The epigenetic landscape of memory CD8^+^ T cells continuously advance during the TEM to TCM transition

**Figure 5.**
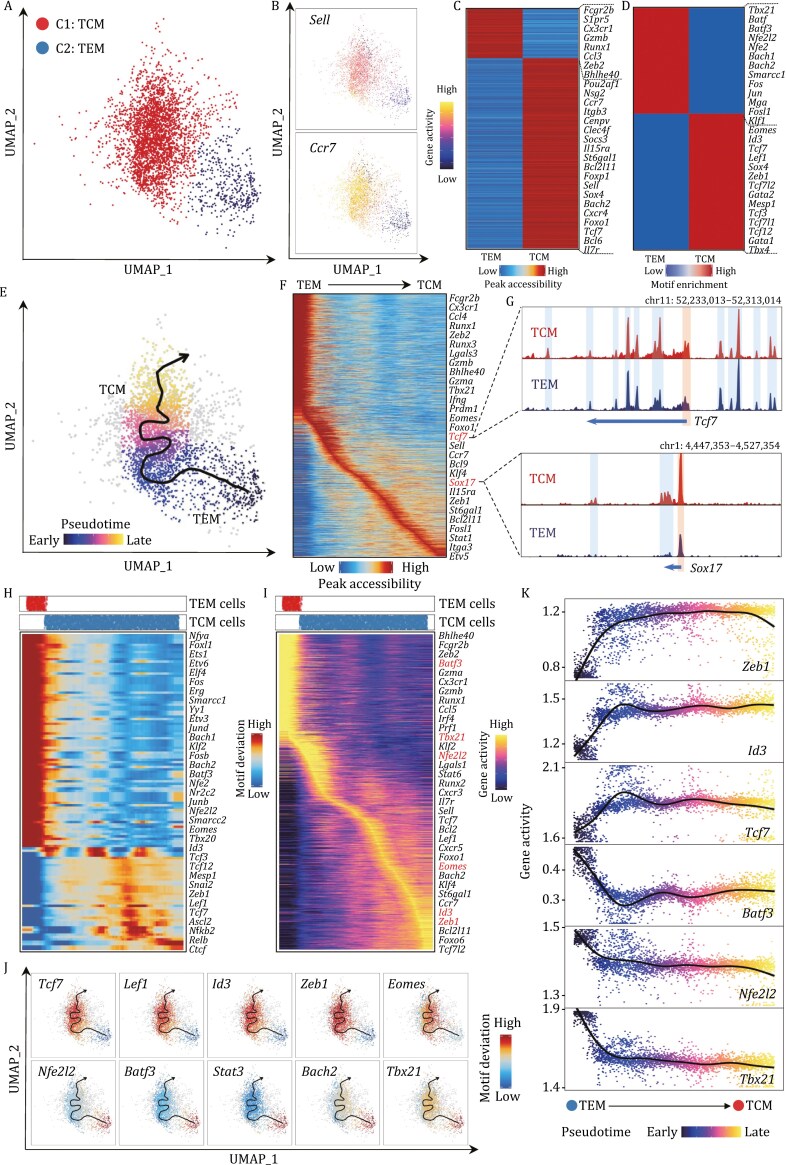
TCM and TEM cells differ in epigenomic landscapes. (A) UMAP projection shows clustered memory CD8^+^ T cells from the D180 sample. Dots represent individual cells and colors indicate cluster identity. (B) UMAP visualization illustrates *Sell* and *Ccr7* gene activity scores. The gene activity score was calculated by the aggregate accessibility of peaks linked to the associated genes, indicating the gene transcription activities. Dot colors based on the log-transformed normalized gene activity scores. (C) Heatmap shows distinct chromatin-accessible peaks between TCM and TEM cells. Notable genes are indicated near their approximate position in the heatmap. (D) Heatmap shows normalized enrichment scores of TF motifs representing distinct chromatin accessible between TCM and TEM cells. Notable TF motifs are indicated near their approximate position in the heatmap. (E) UMAP visualization displays the inferred differentiation trajectory from TEM to TCM cells. UMAP embedding overlaid with pseudotime values of TEM and TCM cells. The smoothed line with the arrow represents the predicted trajectory path. (F) Heatmap shows the dynamic chromatin accessibility along the pseudotime in the differentiation trajectory from TEM to TCM cells. (G) Genome track view visualizes the chromatin accessibility at *Tcf7* and *Sox17* loci by aggregating scATAC-seq signals from each cell type. Promoters and cis-regulatory elements are highlighted in boxes as indicated. (H and I) Heatmap shows TF motif deviation (H) and gene activity (I) scores along pseudotime of the differentiation trajectory from TEM to TCM cells. Notable motifs and genes are indicated near their approximate positions in the heatmaps. The top panel denotes the cell distribution along the pseudotime trajectory from each cell type. (J) UMAP visualization displays motif deviation scores of representative TFs over pseudotime of the differentiation trajectory from TEM to TCM cells. The smoothed line with the arrow represents the predicted trajectory path. (K) Scatter plot shows ordered gene activity scores of representative genes over pseudotime of the differentiation trajectory from TEM to TCM cells. A smoothing line represents loess regressions for gene activity scores. Each dot represents an individual cell colored with pseudotime score.

Memory CD8^+^ T cells comprise TCM and TEM subsets (Sallusto et al., 2004). These subsets differ in terms of migratory patterns, effector functions, proliferative potential, and functional surface markers, such as CCR7 and CD62L (Ahmed and Gray, 1996). However, it remains unclear whether the TCM and TEM subsets can be specified at epigenetic levels other than using surface markers CCR7 and CD62L by flow cytometry. Thus, we reclustered D180 cells in the UMAP embedding space, annotating them into TCM and TEM subsets with the differential gene activity levels of *Sell* and C*cr7* (Figs. 5A, 5B and S6A). The TCM and TEM subsets showed distinct gene activity levels of *Zeb1*, *Foxo1*, *Eomes*, *Klrg1*, *Gzmb*, and *Tbx21*, consistent with the gene expression data (Fig. S6B and S6C). Additionally, we detected 4043 differentially accessible *cis*-elements that exhibited cell type-specific accessibility (Figs. 5C and S6D; Table S8) and enriched different TF binding motifs (Figs. 5D, S6E and S6F). Collectively, our findings indicated that TCMs and TEMs are distinct memory T-cell subsets not only in their expression levels of marker genes but also in their epigenetic properties.

A previous study showed that in the absence of antigen stimulation, a subset of TEM cells could convert into TCM cells (Wherry et al., 2003). However, the underlying epigenetic mechanism governing this inter-subset conversion remains unknown. Thus, we inferred the pseudotime trajectory from TEMs to TCMs to identify critical TFs and their associated *cis*-elements for such fate conversion (Fig. 5E). Next, we revealed that the well-established marker genes for TEM and TCM subsets were largely associated with *cis*-elements showing dynamic accessibility patterns across the trajectory. For example, *cis*-elements linked with *Zeb2*, *Gzmb*, *Gzma*, and *Ifng* represented apparent accessibility in the early pseudotime trajectory, followed by opening *cis*-elements associated with *Prdm1* and *Eomes*. Conversely, except for well-known TFs (*Tcf7*, *Eomes*, Id*3*, and *Zeb1*), novel factors such as *Sox17* are primarily linked by *cis*-elements with accessibility in the late pseudotime trajectory (Fig. 5F and 5G). Motif enrichment and gene activity concordantly showed that *Batf3* primarily emerged in TEM, while *cis*-elements associated with critical TFs for TCM cell fate determination (*Tcf7*, Id*3*, and *Zeb1*) uniquely existed in the late TCM trajectory (Fig. 5H–J). Consistently, we observed a steady increase in the gene activity of *Tcf7,* Id*3, and Zeb1,* and a steady decrease in the gene activity of *Nfe2l2, Batf3,* and *Tbx21* (Figs. 5K and S6G). These results suggested that these TFs associated with dynamic *cis*-elements might maintain the unique integrity of TEM and TCM subsets.

A previous study indicated that differential expression levels of *Cx3cr1* enable two distinct subsets within the TEM population (Gerlach et al., 2016). Indeed, we uncovered that *Cx3cr1* showed varying degrees of chromatin accessibility within TEM cells (Fig. S6H). Among the TEM heterogeneous population, cells exhibiting intermediate chromatin accessibility in *Cx3cr1* aligned close to the TCM population in the developmental trajectory, consociating a wide usage of motifs for *Tcf7*, Id*3*, and *Zeb1,* but an inclusive depletion of motifs for *Nrf2*, *Batf3,* and *Bach2* (Fig. S6I).

Overall, we defined the epigenetic reprogramming of TEMs to TCMs during the memory formation of CD8^+^ T cells.

### Dynamic enhancer–promoter chromatin interactions during memory CD8^+^ T-cell formation

The scATAC-seq approach identifies genome-wide cell type-specific *cis*- and transcription factor-mediated regulation elements. However, which genes are targeted by the identified *cis*-regulatory elements (CREs) remains unresolved because CREs are usually putatively assigned to their nearest genes in bioinformatics analyses. Additionally, it is important to characterize CREs that play essential roles in gene regulation from nonfunctional “parking spots”. Thus, we applied the ChIA-PET approach to map genome-wide RNAPII-mediated enhancer-promoter chromatin interactions between enhancers and their target genes.

**Figure 6.**
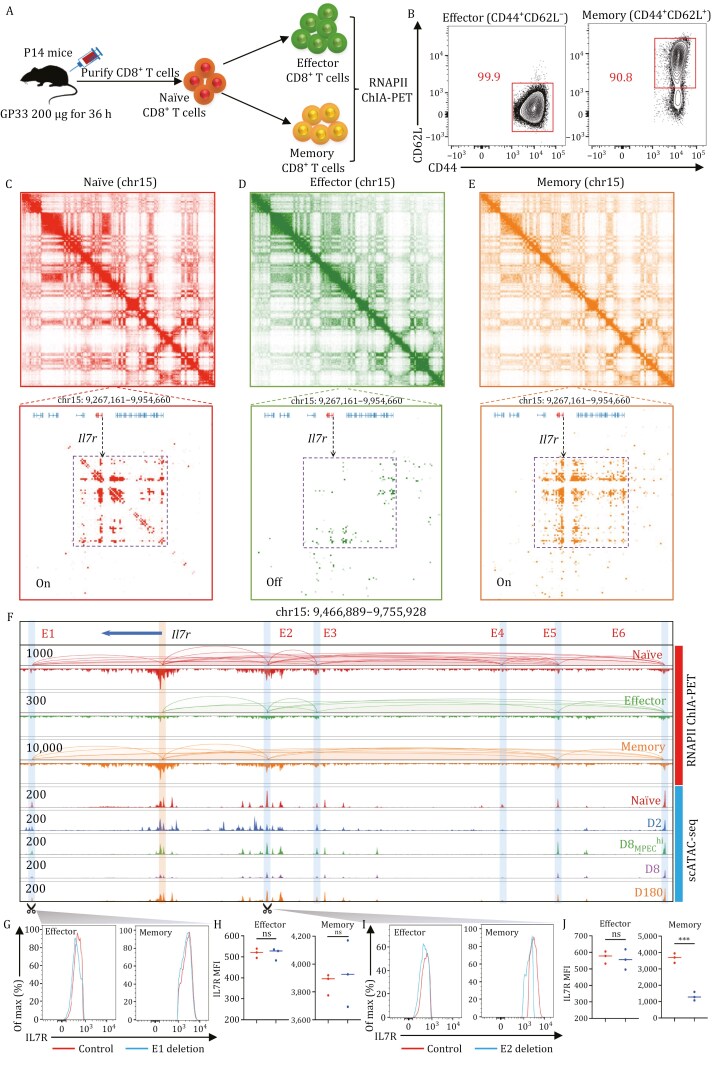
Dynamic enhancer-promotor chromatin interactions during CD8^+^ T-cell differentiation. (A) Schematic overview of the experimental setup and sample collections for RNAPII ChIA-PET sequencing. P14 cells were activated by intravenously injecting 200 μg GP33–41 peptide. After 36 h of activation, mice were sacrificed, and cells from the spleen and lymph nodes were harvested. The cells were then washed and cultured in fresh tissue-culture flasks in a medium supplemented with 20 ng/mL rIL-2 or 20 ng/mL rIL-15 and 1 ng/mL rIL-2. Media supplemented with cytokines were replaced every two days. All ChIA-PET experiments were done using cells cultured for 9 days. (B) Representative flow cytometry contour plot shows the phenotype of *in vitro* differentiated effector and memory CD8^+^ T cells. (C–E) 2D contact map views RNAPII-mediated chromatin interactions on chromosome 15 (top) and a zoomed-in region of *Il7r* (bottom) from naïve (left, C), effector (middle, D), and memory (right, E) CD8^+^ T cells. The ‘On’ and ‘Off’ labels in the left corner of the bottom panels indicated the gene activities according to RNAPII looping activities at the *Il7r* locus. (F) Genome browser views chromatin loops and binding peaks detected by RNAPII ChIA-PET sequencing for naïve (red), effector (green), and memory (orange) CD8^+^ T cells, and aggregated scATAC-seq signals of each sample at the *Il7r* locus. The promoter peak was highlighted in light orange, and the detected interaction enhancers were highlighted in light blue. (G–J) The expression level of IL7R in effector and memory CD8^+^ T cells after E1 (G and H) and E2 (I and J) enhancer deletions.

We performed RNAPII ChIA-PET experiments in naïve, effector, and memory CD8^+^ T cells (Fig. 6A and 6B). After applying stringent quality control (Fig. S7A and S7B), 2D contact maps showed that the overall chromatin interactions between different samples were highly comparable (Fig. S7C and S7D), consistent with the fact that most CREs (56.31%) possessed constitutive accessibility after T-cell activation (Fig. S1J). However, prominent cell state-specific enhancer-promoter chromatin interactions remained in different T-cell subsets. For instance, the persistent expression of IL7R is a cardinal feature of naïve and memory CD8^+^ T cells, but IL7R is only expressed by a small fraction of effector CD8^+^ T cells during acute LCMV-Arm^+^ infection, consistent with the accessibility profile at the *Il7r* locus (Fig. 3J). RNAPII ChIA-PET data revealed that the *Il7r* TSS was intensively connected with multiple distal enhancers in naïve and memory cells. In contrast, those RNAPII-mediated chromatin interactions were strikingly absent in effector cells (Fig. 6C–F). The *Cd44* and *Eomes* genes also showed coordinated RNAPII-mediated chromatin interaction patterns with their chromatin accessibility profiles (Fig. S7E–H). Our ChIA-PET data indicated that the changes in RNAPII-mediated chromatin looping precisely accorded with the dynamics of chromatin accessibility detected by scATAC-seq data. More strikingly, in memory cells, the signature genes for the effector phenotype, such as *Ifng* and *Gzmb*, were involved in strong chromatin interactions with distal enhancers forming chromatin hubs, which might provide the epigenetic regulation basis for the rapid recalling response when memory cells re-encounter pathogens (Fig. S8A–D).

To validate the function of enhancers identified by RNAPII ChIA-PET data, we chose two enhancers (E1 and E2, Fig. 6F) at the *Il7r* locus, in which E2 displayed a stronger chromatin interaction with the *Il7r* promoter than E1 in both naïve and memory cells. After transducing the Cas9-expressing CD8^+^ T cells with a pair of single-guide RNAs (sgRNAs) or control vector flanking E1 or E2, cells were then cultured *in vitro* for ten days with IL7 and IL15 to favor memory differentiation, we observed that the depletion of enhancer E1 does not alter the expression of CD127 (*Il7r*) in effector and memory CD8^+^ T cells (Fig. 6G and 6H). However, the depletion of enhancer E2 caused a noticeable reduction in CD127 (*Il7r*) expression compared with that of the vector in memory CD8^+^ T cells but not in effector CD8^+^ T cells (Fig. 6I and 6J). These results indicated that enhancers regulated gene activity in a combinatory manner via long-range chromatin interactions but possessed distinct attributes in gene activation.

Collectively, the ChIA-PET data unfolded that enhancers are organized into chromatin interaction hubs that regulate distinct gene activities, coordinating the epigenetic reprogramming of chromatin accessibility during CD8^+^ T-cell differentiation.

### Validation of crucial TFs for memory T-cell differentiation

**Figure 7.**
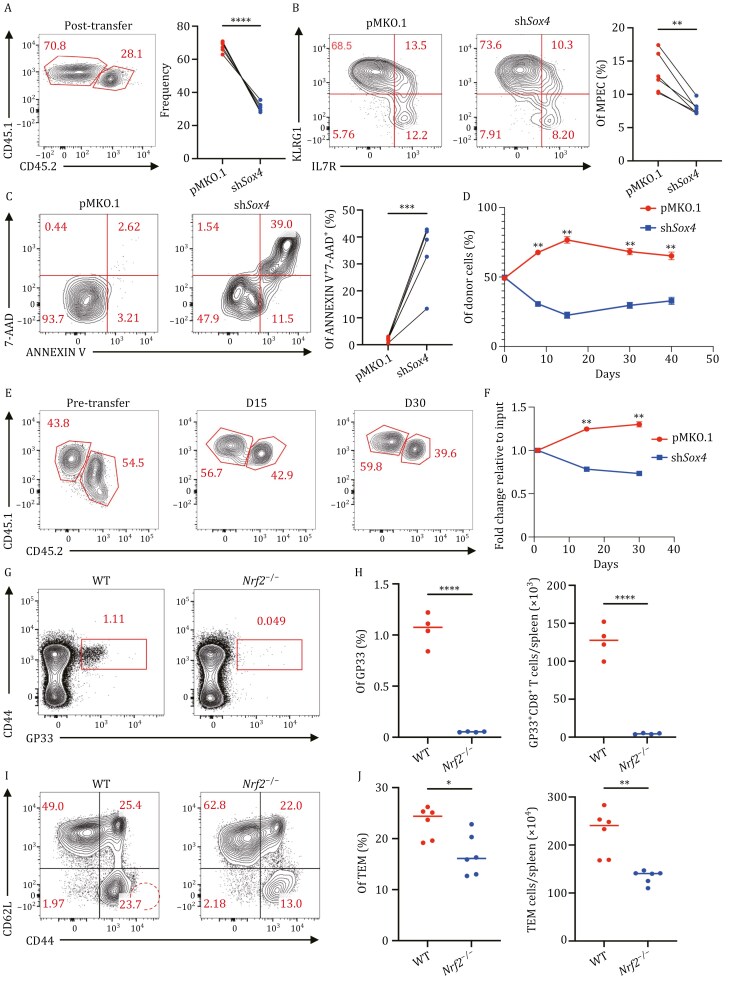
Functional validations of *Sox4* and *Nrf2* for memory CD8^+^ T-cell differentiation. (A) Congenic distinct CD8^+^ P14 cells were transduced with retrovirus encoding short hairpin RNA (shRNA) targeting *Sox4* (sh*Sox4*, CD45.1^+^CD45.2^+^) or vector (pMKO.1, CD45.1^+^), the GFP^+^ cells were sorted and then 1:1 cotransferred into CD45.2^+^ recipient mice, followed by infection with LCMV-Arm^+^, the differentiation of CD8^+^ T cells were monitored (left). Frequency of sh*Sox4* versus control (pMKO.1) in the spleen on D8 post-LCMV-Arm^+^ infection (right). (B) Representative flow cytometry plots show KLRG1 versus IL7R from D8 LCMV-Arm^+^ infection (left). Enumeration of MPEC cells from LCMV-Arm^+^ infection as gated in the left panel (right). (C) Representative flow cytometry plot shows 7-AAD versus ANNEXIN V from D8 LCMV-Arm^+^ infection. Cells were gated on MPECs (left). Enumeration of ANNEXIN V^+^7-AAD^+^ cells from LCMV-Arm^+^ infection as gated in the left panel (right). (D) Frequency of sh*Sox4* versus control (pMKO.1) over time in the spleen in LCMV-Arm^+^ infection. Data are presented as mean values ± SEM. (E) On D8 post-LCMV-Arm^+^ infection, MPEC cells were sorted and cotransferred into infection-matched mice. The frequency of sh*Sox4* (CD45.1^+^CD45.2^+^) versus pMKO.1 (CD45.1^+^) was checked over time. (F) Line plot shows the fold change relative to input cells among different time points between pMKO.1 and sh*Sox4* MPEC cells, Data are presented as mean values ± SEM. (G) *Nrf2*^−/−^ mice and their wild-type (WT) littermates were infected with LCMV-Arm^+^ and sacrificed on D40 postinfection. GP33 tetramer was used for detecting antigen-specific CD8^+^ T cells. Representative flow cytometry plots on 40-days post-LCMV-Arm^+^ infection. (H) The dot plot shows the percentage (left) and absolute number (right) of GP33^+^CD8^+^ T cells between WT and *Nrf2*^−/−^ mice, the line represents mean values. (I) *Nrf2*^−/−^ mice and their wild-type (WT) littermates were infected with Lm-OVA and sacrificed on D40 postinfection. OVA257-264 tetramer was used for detecting antigen-specific CD8^+^ T cells. Representative flow cytometry plots on 40-days post-Lm-OVA infection. (J) The dot plot shows the percentage (left) and absolute number (right) of OVA257-264 specific CD8^+^ T cells between WT and *Nrf2*^−/−^ mice, the line represents mean values.

MPECs exhibit an enhanced ability to survive and differentiate into *bona fide* memory T cells. Our scATAC-seq data have indicated that *Sox4* represented the synchronized patterns of gene activity and motif enrichment in MPECs (Figs. 4K and S5I). Furthermore, previous studies have revealed that *Sox4* regulates stem cell differentiation and multiple developmental pathways (Cheung et al., 2000; Sinner et al., 2007). *Sox4* also regulates T-cell differentiation in the thymus and the recall ability of memory T cells (Hu and Chen, 2013; Schilham et al., 1996, 1997). However, whether *Sox4* might be essential for developing MPECs remains to be determined. Toward this end, we transduced congenic distinct P14 CD8^+^ T cells with vector or shRNA targeting *Sox4* and transferred them into mice at a 50/50 ratio, followed by infection of the recipients with LCMV-Arm^+^. Knockdown of *Sox4* in MPECs resulted in a 70% reduction in the expression level compared with that in the cells transduced with a control vector (Fig. S9A). Flow cytometry analysis of CD8^+^ T cells on Day 8 after infection showed a significantly lower frequency of the MPEC subset among sh*Sox4*-transduced T cells than among vector-transduced T cells (Fig. 7A and 7B), which might be related to enhanced apoptosis in sh*Sox4*-transduced MPECs (Fig. 7C). BIM/BCL2 balance is critical for maintaining T cell homeostasis (Wojciechowski et al., 2007), we checked BCL2 and BIM expression, and both BCL2 and BIM are higher in sh*Sox4*-transduced MPECs than the vector-transduced MPECs. However, the BCL2/BIM ratio is reduced in sh*Sox4-*transduced MPECs (Fig. S9B and S9C), which may contribute to faster apoptosis. Kinetic analysis of sh*Sox4*- and vector-transduced T cells from the spleen showed that sh*Sox4*-transduced cells exhibited sharply decreased expansion but slight recovery from Days 15 to 40 postinfection. However, the percentage of sh*Sox4*-transduced T cells was persistently lower than that of vector-transduced T cells (Fig. 7D). These results indicate that *Sox4* governs MPEC differentiation and might influence the transition of MPECs to memory T cells.

To investigate the role of *Sox4* in MPEC-to-memory T-cell transition, we sorted vector- or sh*Sox4*-transduced MPECs in D8 cells and then cotransferred them into infection-matched mice. The percentage of sh*Sox4*-transduced cells decreased over time, indicating that *Sox4* knockdown impacts not only MEPC formation but also the MPEC-to-memory T-cell transition (Fig. 7E and 7F). In addition, the reduction in MPECs was even more significant when OT-1 CD8^+^ T cells were transduced with sh*Sox4* than when they were transduced with empty vector (Fig. S9D–F), while the percentage of SLECs was significantly higher in sh*Sox4*-transduced OT-1 cells than in control OT-1 cells (Fig. S9E).

Our results suggested that *Sox4* played a vital role in fostering MPECs and their further differentiation into mature memory cells. Therefore, we compared genetic differences between vector- or sh*Sox4*-transduced MPECs by bulk RNA-seq. The memory-associated genes *Tcf7*, *Eomes*, *Bhlhe40*, *Prdm1*, and *Bcl2* are enhanced in sh*Sox4*-transduced MPECs but not vector transduced MPECs (Fig. S9G), which may explain the slight recovery from Days 15 to 40 postinfection (Fig. 7D). The higher expression of TCF1 and EOMES in sh*Sox4*-transduced MPEC cells were also validated by flow cytometry (Fig. S9H–K).

Next, we focused on nuclear factor erythroid-derived 2-like 2 (*Nfe2l2*, *Nrf2*), which showed both higher motif enrichment and gene activity in TEM than in TCM (Fig. 5H and 5I). *Nrf2* is a ubiquitously expressed transcription factor regulating multiple stress-responsive pathways that protect against oxidative stress and maintain protein and metabolic homeostasis(Merchant et al., 2011). *Nrf2* has also been reported to participate in T-cell differentiation and NKT cell development (Pyaram et al., 2019). However, the role of *Nrf2* in regulating memory CD8^+^ T-cell development remains unknown. *Nrf2*^−/−^ mice and their wild-type littermates were infected with LCMV-Arm^+^ or Lm-OVA and sacrificed on Day 40 postinfection. Indeed, we observed that the percentage and absolute number of antigen-specific memory CD8^+^ T cells decreased dramatically in the spleen (Fig. 7G–J), as did the percentage and absolute number of TEMs (Fig. S9L and S9M).

Collectively, we validated that *Sox4* and *Nrf2* identified by our TF enrichment analysis essentially regulate memory CD8^+^ cell differentiation, in which *Sox4* promoted differentiation of the MPEC subset and *Nrf2* subsequently established effector memory T-cell development.

## Discussion

In this study, our genome-wide scATAC-seq and ChIA-PET analysis of CD8^+^ T-cell subsets during acute viral and bacterial infection demonstrated that the acquisition of cell state-specific functional and phenotypic properties upon memory T-cell differentiation is a well-coordinated process underpinned by distinct chromatin accessibility, dynamic promoter-enhancer interactions, and various critical TFs.

Naïve antigen-specific CD8^+^ T cells encounter cognate antigens and differentiate into a plastic, reprogrammable chromatin state to enable the differentiation of distinct T cell subsets. The *cis*-element changes are most striking in earlier activated cells (D2), consistent with the previous finding that the differentially expressed genes peaked within 48 h of infection (Best et al., 2013) and indicated that the epigenomic program of memory is mainly preloaded early after infection. Furthermore, even on D180, further chromatin state remodeling is also detected, accompanied by the TEM to TCM transition. This long stepwise fashion in epigenomic alterations may provide sufficient time for these cells to fully acquire polyfunctionality and stemness. The TF deviation coexpression network analysis revealed key TFs that ensure appropriate gene expression at distinct stages of the differentiation process. First, the *Tcf7*- and *Lef1*-centered transcriptional regulatory networks are critical for maintaining a steady state, consistent with the findings of a previous report (Xing et al., 2016). Then, after naïve T cell activation on D2, *Batf* and *Batf3* interact downstream of TCR signaling, *Fos*, *Jun*, and *Fli1*, which is required for CD8^+^ T-cell expansion and differentiation (Kuroda et al., 2011; Z. Chen et al., 2021). However, following continued antigen stimulation, T cells proceed to a fully differentiated state driven by the activity of *Tbx21* and *Prdm1* (Joshi et al., 2007; Rutishauser et al., 2009). Finally, a few effector T cells survive and progress to memory T cells, in which *Zeb1* and Id*3* synchronize with *Tcf7* and *Lef1* to maintain the homeostasis and recall potential of memory T cells (Shan et al., 2022). Combined with cell state-specific TF motif enrichment, we further investigated the interaction pattern between TFs and their motifs. Interestingly, although most of the TFs emerge congruently with their motif enrichments, we found unsynchronized patterns between TF activities and their motif enrichments in the differentiation process, which could occur via multiple mechanisms, including TF localization, cofactor availability, transcriptional velocity, and the posttranslational modification of TFs. This special temporal regulation adds complexity to the gene regulation process and might provide a unique window for finetuning the immune response during CD8^+^ T-cell differentiation.

The differentiation trajectory of memory CD8^+^ T cells has been debated for a long time. One model states that memory CD8^+^ T cells are the progeny of a subset of effector CD8^+^ T cells (MPECs) (Ahmed et al., 2009; Youngblood et al., 2013, 2017); another distinct model states that memory cell fate is determined after the first cell division, bypassing the effector stage (Ahmed et al., 2009; Arsenio et al., 2014; Pais Ferreira et al., 2020). In the current study, our scATAC-seq data showed that even earlier activated cells (D2) exhibited exclusively higher granzyme B chromatin accessibility and gene activity than naïve cells, which demonstrated that all the cells had presumably undergone an effector stage. Furthermore, the pseudotime trajectory analysis revealed that all memory CD8^+^ T cells originated from effector cells rather than representing a different cell lineage arising from naïve cells. Additionally, we revealed a subpopulation of cells named EECs in this study, with the KLRG1^lo^CD127^lo^ phenotype on Day 8 postinfection as the progenitor for developing MPECs and SLECs. The bifurcating differentiation from EECs is determined by essential TFs in a distinct dichotomous manner, in which hallmark TFs, including *Tcf7*, *Zeb1*, Id*3*, and *Sox4*, drive the development of MPECs, while higher expression levels of *Tbx21*, *Runx3*, and *Zeb2* dictate SLEC cell fate. Together, these data together support the notion that memory CD8^+^ T cells differentiate from effector cells early upon acute viral infection.

The dynamic organization of chromatin architecture is pivotal for many important molecular events, including the spatiotemporal regulation of the transcription of cell type-specific genes, which is critical for cell fate decisions and lineage specification (Kragesteen et al., 2018; Russ et al., 2023; Stadhouders et al., 2018). Our genome-wide ChIA-PET data analysis allowed a broad, unbiased stratification of the scATAC-seq data, providing a systemic functional interaction map between distal enhancers and promoters. Our *Il7r* distal enhancer deletion experiment demonstrated that cell state-specific enhancers could be an optimal way to precisely manipulate T cells after viral infection or vaccination.

We also validated the functions of two TFs, *Sox4* and *Nrf2*, identified by scATAC-seq data, confirming their respective essential roles in differentiating MPEC and TEM subsets. *Sox4* is critical for regulating *Tbx21*, *Eomes*, and *Tcf7*, enhancing the recall response of memory CD8^+^ T cells (Hu and Chen, 2013). However, the role of *Sox4* in developing memory CD8^+^ T cells, especially MPECs, has not been reported. Here, we validated that *Sox4* is required for the formation of MPECs and the subsequent transition from MPECs to memory T cells. Providing new insight into the functional role of *Sox4* and a potential strategy for manipulating memory T-cell differentiation. While our data suggest that *Sox4* is involved in maintaining the survival and proliferation of MPECs, the precise molecular mechanism by which *Sox4* regulates MPEC differentiation remains incomplete. Further studies, including chromatin immunoprecipitation sequencing (ChIP-seq) or more targeted functional assays, will be necessary to dissect the direct downstream targets of *Sox4* and their roles in MPEC development. *Nrf2* is a critical regulator of the antioxidant glutathione in response to stimulation. *Nrf2*-deficient dendritic cells have enhanced costimulatory receptor expression and increased antigen-specific CD8^+^ T-cell stimulation capacity (Yeang et al., 2012). It is unclear whether *Nrf2* deficiency could influence memory CD8^+^ T-cell formation. Here, we demonstrated that *Nrf2* is essential for memory T-cell differentiation, especially TEM formation, while the data strongly suggest a critical role for *Nrf2* in TEM, future studies using tissue- or cell-specific knockout models will be necessary to dissect the direct, cell-intrinsic functions of *Nrf2* in memory T-cell development.

Despite the successful construction of the single-cell epigenomic landscape and high-resolution enhancer–promoter interaction map during the development of memory CD8^+^ T cells, further dissecting and validating the cell state-specific enhancers on important TFs could considerably maximize the value of our data. This study also adds to a growing appreciation of how chromatin accessibility (1D) and chromatin architecture (3D) underpin the regulation of gene expression within T cells. Enhancing the quality and increasing the quantity of the memory CD8^+^ T-cell pool has been an essential rationale in designing vaccines based on cellular immunity. Thus, an in-depth study of the characteristics and molecular mechanisms of memory CD8^+^ T cells will enable us to better understand the nature of immunological memory and provide insights into both basic T-cell biology and translational immunotherapy.

## Materials and methods

### Mice

CRISPR/Cas9 knock-in mice, CD45.1^+^ and CD45.2^+^ congenic mice (B6.SJL-Ptprc^a^ Pepc^b^/BoyJ), and OT-1 transgenic mice (expressing a TCR specific for the OVA257-264 peptide in the context of H-2K^b^) were purchased from the Jackson Laboratories. P14 transgenic mice (carrying a transgenic T-cell antigen receptor that recognizes the H-2D^b^ GP33–41 epitope of LCMV) were a gift from Dr. Rafi. Ahmed (Emory University). *Nrf2*^−/−^ mice were gifted from Prof. Lanfen Chen from Xiamen University. CRISPR/Cas9 knock-in P14 mice were generated by crossing CRISPR/Cas9 knock-in mice with P14 mice. All the mice were kept on a C57BL/6J background and used at 6–8 weeks. All mice were housed in specific pathogen-free (SPF) conditions. All mouse experiments were performed by the guidelines of the Institutional Animal Care and Use Committees of the Third Military Medical University.

### Infection model

The lymphocytic choriomeningitis virus Armstrong (LCMV-Arm^+^) was kindly provided by Dr. Rafi. Ahmed at Emory University and propagated in the lab. Generally, mice were intraperitoneally infected (i.p.) with 2 × 10^5^ PFU (plaque-forming units) Armstrong. For the sorting of P14 cells on Day 2 postinfection, mice were intravenously infected with 2 × 10^6^ PFU Armstrong. Attenuated *Listeria monocytogenes* expressing OVA (Lm-OVA) was a gift from Dr. Xinyuan Zhou (Third Military Medical University) and was generated by introducing an in-frame deletion in the actA gene as described (Zhao et al., 2010). Bacteria were grown overnight in brain heart infusion (BHI) media and quantified by optical density (OD) (1 OD refers to 8 × 10^8^ CFU [colony-forming units]). Mice were intravenously infected (i.v.) with 1 × 10^5^ CFU Lm-OVA. All mouse experiments and breeding conditions were performed following the guidelines of the Institutional Animal Care and Use Committees of the Third Military Medical University.

### Adoptive transfer of T cells, cell sorting, and scATAC-seq library generation

For the LCMV-Arm^+^ infection model, CD45.1^+^ pMKO.1 vector or CD45.1^+^CD45.2^+^ shRNA targeting *Sox4* transduced P14 were 1:1 mixed, and 1 × 10^5^ cells were transferred into the CD45.2^+^ recipients followed by 2 × 10^5^ PFU of LCMV-Arm^+^ infection. This experimental setup is critical as it allows both cell populations to experience the same *in vivo* environment, including identical exposure to antigens, cytokines, and other factors influencing activation, proliferation, and differentiation. On D8, vector transduced MPECs were sorted as L/D^−^CD44^+^CD8^+^CD45.1^+^CD127^+^KLRG1^−^, *shSox4* transduced MPECs were sorted as L/D^−^CD44^+^CD8^+^CD45.1^+^CD45.2^+^CD127^+^KLRG1^−^. For the Lm-OVA infection model, 1 × 10^5^ CD45.1^+^ pMKO.1 vector or CD45.1^+^ shRNA targeting *Sox4* transduced OT-1 were separately transferred into CD45.2^+^ recipients followed by 1 × 10^5^ CFU of Lm-OVA infection.

For cell sorting, naïve CD8^+^ T cells were purified from CD45.1^+^ P14 or CD45.1^+^ OT-1 splenocytes. For effector and memory CD8^+^ T cell sorting, 5 × 10^4^ P14 or OT-1 CD8^+^ T cells were transferred into congenic distinct CD45.2^+^ mice by intravenous (i.v.) injection and then infected with 2 × 10^5^ PFU of LCMV-Arm^+^ intraperitoneally for P14 or 1 × 10^5^ CFU of Lm-OVA intravenously for OT-1, respectively 1 day later. For experiments where P14 or OT-1 CD8^+^ T cells were collected 2 days after infection. To enable the isolation of sufficient numbers of cells for scATAC-seq analysis, 1 × 10^6^ P14 or OT-1 CD8^+^ T cells were transferred and then infected with 2 × 10^6^ PFU of LCMV-Arm or 1 × 10^6^ CFU of Lm-OVA intravenously to ensure robust infection establishment and rapid recruitment of transferred cells into the immune response. Transfer of a large number of cells and change the routine of infection can alter the magnitude and kinetics of the immune response and therefore might represent a caveat of this study. On the indicated day, the spleen was mashed through a 70 mm nylon cell strainer (BD Falcon). Red blood cells were lysed with a hypotonic ammonium–chloride–potassium (ACK) buffer. Naïve T cells were sorted as L/D^−^CD44^−^CD8^+^, effector cells were sorted as L/D^−^CD45.1^+^CD44^+^CD8^+^ on Days 2 and 8 postinfection, memory T cells were sorted as L/D^−^CD45.1^+^CD44^+^CD8^+^ on Day 180 postinfection.

RNA-seq libraries were constructed by SMART-Seq V4, then assessed by lllumina NovaSeq 6000. scATAC-seq libraries were generated using the 10x Genomics Chromium Cell ATAC Reagent Kit (v1). Briefly, approximately 50,000 sorted P14 or OT-1 cells were used for the nuclei preparation according to the manufacturer’s instructions. Then, 15,000 nuclei were loaded into a 10× Chromium controller. All library preparation was performed according to the manufacturer’s instructions. All scATAC-seq libraries were further assessed using an Agilent Tapestation and quantified using a KAPA Library Quantification Kit (KK4824) and then sequenced on an Illumina NovaSeq 6000.

### shRNA-mediated knockdown and retroviral transduction

The detailed procedure was done as previously described (He et al., 2016). Briefly, 293T cells were plated in 10 cm dishes 1 day before transfection and individually transfected with 12 µg of each shRNA retroviral construct and 6 µg of PCL-Eco using TransIT-293 Transfection Reagent (Mirus). The retroviral supernatant was collected 48 hours after transfection, *in vivo* activated P14 or OT-1 T cells were transduced with retrovirus encoding knockdown shRNA and control retrovirus separately. To activate P14 T cells *in vivo*, P14 mice were intravenously injected with 200 μg GP33–41 peptide. Eighteen hours later, activated CD8^+^ T cells were isolated, purified, and transduced. OT-1 cells were activated by anti-CD3 (1 μg/mL) and CD28 (1 μg/mL) for 36 h. The hairpin sequence for shRNA was as follows: 5′-CCGGGCGAGATGATCTCGGGAGATTCTCGAGAATCTCCCGAGATCATCTCGCTTTTTG-3′.

### *In vitro* effector and memory T-cell differentiation

The effector and memory T cells were induced as previously described with minor modifications (Manjunath et al., 2001). Briefly, 200 μg GP33–41 peptide was intravenously injected into the P14 mice. After 36 hours of activation, mice were sacrificed, and cells from the spleen and lymph node were harvested. The cells were then washed and cultured in fresh tissue-culture flasks in a medium supplemented with 20 ng/mL rIL-2 or 20 ng/mL rIL-15 and 1 ng/mL rIL-2. Media supplemented with cytokines were replaced every 2 days. All experiments were done using cells cultured for 9 days.

### CRISPR-Cas9-mediated enhancer deletion of CD8^+^ T cells

The regions at upstream 57 kb (E1) and downstream 45 kb (E2) from the *Il7r* TSS were deleted using two pairs of sgRNA targeting sequences flanking each region. Briefly, a retroviral vector with a tandem expression of two sgRNAs from independent hU6 promoters was developed by modifying pSL21-mcherry vectors (Addgene, 164410). CD8^+^ T cells from CRISPR/Cas9 knock-in P14 mice were transduced with a pair of targeting sgRNAs or negative control sgRNAs. Cells were sorted based on mcherry expression, and genomic DNA was isolated using QIAamp DNA Micro Kit according to the manufacturer’s instructions. PCR was performed on genomic DNA from each sample using the deletion screening primer pairs and run on 2% agarose gel. Depletion was verified by submitting the PCR amplification products for sanger sequencing.

### Preparation of single-cell suspensions from mouse samples

Splenocyte isolation was acquired as previously described (Liu et al., 2022). In brief, Spleens were resected with sterilized scissors and crushed with the blunt part of a 1 mL syringe on Petri dishes containing 2 mL red blood cell lysis buffer. One minute later, 5 mL R2 medium was added to neutralize the red blood cell lysis buffer. The resulting cell suspensions were filtered through a 70 μmol/L filter into a 15 mL conical tube, centrifuged at 1,800 rpm for 6 min at 4°C, and the supernatants were discarded. Cells were resuspended in 3 mL of R2 medium (RPMI-1640 supplemented with 2% FBS).

### Flow cytometry

Flow cytometric analysis was performed on FACSCanto II or FACSFortesa instruments (BD Biosciences). Surface staining was performed in PBS containing 2% bovine serum albumin or FBS (*w*/*v*). Approximately 0.5–1 × 10^6^ cells for each sample were stained with surface antibody cocktails for 30 min on ice.

### RNA Pol II *in situ* ChIA-PET library preparation and sequencing

RNA Pol II *in situ* ChIA-PET was performed as previously described (Wang et al., 2021) with modifications. Briefly, ∼100–200 million cultured primary T cells were harvested and fixed with 45 mL of 1.5 mmol/L ethylene glycol-bis (succinic acid N-hydroxysuccinimide ester) in DPBS buffer for 20 min at room temperature (RT) with 15 rpm rotating. A final concentration of 1% formaldehyde was added directly and followed by a rotation with 15 rpm at room temperature for 15 min. Before washing, quenched the crosslinked sample by adding a final concentration of 125 mmol/L glycine at 15 rpm rotating for 10 min at RT. Collected all cell pellets with 1000 rcf for 5 min at 4°C and washed the cell twice with 45 mL PBS before harvesting the cells. The dual crosslinked cells were aliquoted into 10 million cells in each 1.5 mL LowBand tube for following library construction or storing at a −80°C freezer.

Cells were lysed using 1 mL 0.1% SDS lysis buffer (50 mmol/L HEPES-KOH pH 7.5, 150 mmol/L NaCl, 1 mmol/L EDTA, 1% Triton X-100, 0.1% sodium deoxycholate, 0.1% SDS, and 1× Complete Mini EDTA-free Protease Inhibitor Cocktail in nuclease-free water) and rotated at 15 rpm at 4°C for 1 h. The cells were then collected and resuspend in 0.55% SDS lysis buffer with 1× protease inhibitor for incubating sequentially at RT for 10 min, 62°C for 10 min, and 37°C for 10 min. The lysed cells were quenched by adding a final concentration of 1.5% Triton X-100 solution and incubated at 37°C for 15 min. The quenched cells were digested overnight at 37°C with 60 U AluI and added 50 μL 10× CutSmart buffer with a final volume of 500 μL and rotated the digestion solution at 35 rpm. After performing A-tailing and proximity-ligation using Klenow large fragment enzyme and T4 DNA ligase and the DNA was proximity ligated by a single biotinylated bridge linker: forward strand: 5′-[Phos]CGCGATATC/iBIOdT/TATCTGACT-3′, reverse strand: 5′-[Phos]GTCAGATAAGATATCGCGT-3′.

The chromatin was obtained and sonicated to generate fragments with an average length of 1–3 kb. The monoclonal antibody against RNA Polymerase II preincubated protein G beads was used to enrich RNAPII-bound chromatin fragments overnight, rotating with 10 rpm at 4°C. The on beads chromatin DNA fragments were washed following four different cold buffers and rinsed in 100 μL TE-1% SDS solution for 1 h at 65°C with 1,100 rpm rotation. The solution was added 150 μL Qiagen EB buffer for another 45 min decrosslinking. Collected all supernatant, added 10 μL proteinase K, and rotated at 900 rpm overnight at 65°C. The DNA fragments were purified with Zymo genomic DNA clean&concentrator kit and eluted with 20 μL nuclease-free water. Proceed with tagmentation for 50 ng ChIP-DNA in each 200 μL PCR tube using 5 μL Tn5 transposase at 55°C for 10 min to obtain a 0.1–1 kb tagmentated DNA profile. Purified the tagmentated DNA with DNA cleanup columns and immobilized with the preblocked and precleaned M280 streptavidin dynabeads in final 1× binding and washing buffer at RT for 45 min with 15 rpm rotation. The supernatant was discarded, and the beads were washed five times with 500 μL prewarmed at 37°C 0.5% SDS/2× SSC buffer, and the beads were rinsed in 30 μL elution buffer. A PCR reaction on beads with 11–13 cycles was performed to construct the *in situ* ChIA-PET libraries, and the PCR products were purified with 1× AMPure beads. These final libraries were then subjected to a 0.6–0.8× double-size selection using AMPure beads and paired-end sequencing (2 × 150 bp).

### Preprocessing of single-cell ATAC-seq data

Raw single-cell ATAC-seq data from each sample was processed using the 10x Genomics Cell Ranger ATAC pipeline (v1.2.0) and mapped to the mouse reference genome (mm10). Accordingly, we aligned the demultiplexed FASTQ reads to the reference genome, called accessible peaks, and quantified the unique molecular identifier (UMI) for each peak in every cell barcode using the “cellranger-atac count” function with default parameters. The preprocessed peak-barcode count matrices were used for initial quality evaluation in R (v4.0.0), Seurat (Hao et al., 2021; Stuart et al., 2019) (v4.0.6), and Signac (Stuart et al., 2021) (v1.1.1) using default parameters unless otherwise noted. First, quality control metrics were calculated in the Signac package to perform quality evaluation (peak_region_fragments >3,000 & <100,000, pct_reads_in_peaks >40, blacklist_ratio <0.01, nucleosome_signal <10, TSS.enrichment >2 or 1.5) for each sample, and then were merged and requantified with the union peak list. The union peak list was called using MACS 2 (Zhang et al., 2008) with “--model --shift -100 --extsize 200” parameters based on all sample bam files and filtered with mouse blacklist regions. The custom union peak list was then added to the Signac object using the “FeatureMatrix” and “CreateChromatinAssay” functions. Second, to eliminate the biological batch effects across different samples, we employed the integration methods adopted by the python package scAND (Dong and Zhang, 2021) to assemble multiple scATAC-seq datasets into an integrated and unbatched one. According to, each sample was processed using the “scAND.Run_scAND” function with “*d* = 50, weights = 0.5” parameter settings, and then performed sparse multiple canonical correlation analysis (CCA) using the “MultiCCA” function with “niter = 200, num. ccs = 5” parameters. Last, the scanpy (Wolf et al., 2018) (v1.6.0) package was applied to compute the neighborhood graph and dimension reduction using the “sc.pp.neighbors” and “sc.tl.umap” functions.

### Analysis of single-cell ATAC-seq data

After quality control and batch effect correction, the preprocessed scATAC-seq data was used for downstream analysis using the ArchR (Granja et al., 2021) (v1.0.1) R package with default parameters unless otherwise noted. In addition to the Signac quality evaluation, further cell filtering was performed in the ArchR to keep only cell barcodes with at least 1000 fragments per cell and a TSS enrichment score ≥ 4. The number of retained cells for each sample was summarized in Fig. S1D. In the end, we obtained a total of 19,950 high-quality CD8^+^ T cells for further analysis. Iterative latent semantic indexing (LSI) dimensionality reduction was performed with the “addIterativeLSI” function (useMatrix = “TileMatrix”, iterations = 4, varFeatures = 15,000, dimsToUse = 1:30), and the top 30 dimensions were selected for downstream analyses. Precalculated uniform manifold approximation and projection (UMAP) dimensional embeddings by scanpy were added to the ArchR object for data visualization in Fig. 1C.

To estimate gene activity in every CD8^+^ T cell, we used the ArchR gene activity scores to calculate with the “addGeneScore” function and then imputed gene activity by the MAGIC (van Dijk et al., 2018) algorithm for UMAP visualization (Figs. 1D and S1E). To get differentially accessible regions (DARs) between different samples, single-cell chromatin accessibility data were used to generate pseudobulk replicates from scATAC-seq datasets using the “addGroupCoverages” and “addReproduciblePeakSet” functions for peak calling with MACS 2 (Zhang et al., 2008). DARs for each sample were calculated using the “getMarkerFeatures” and “getMarkers” functions based on the called peak matrix (useMatrix = ‘PeakMatrix’, bias = c (‘TSSEnrichment’, ‘log10[nFrags]’), testMethod = ‘Wilcoxon’, cutOff = ‘FDR <= 0.05 & Log2FC >= 1’), and visualized the accessibility profiles of the DARs by ‘plotMarkerHeatmap’ function in Fig. 1F. The obtained DARs in each sample were annotated using the “annotatePeak” function by ChIPseeker (Yu et al., 2015) R package in Fig. S1H and analyzed for TF motif enrichment using the “addMotifAnnotations” and “peakAnnoEnrichment” functions with “motifSet = ‘cisbp’, cutOff = ‘FDR <= 0.05 & Log2FC >= 1’” parameter settings in Fig. 2A.

### TF motif deviation enrichment analysis

TF motif deviation enrichment for peaks in each cell was calculated using the ChromVAR (Schep et al., 2017) R package. Briefly, a background peak set controlling for total accessibility, and GC content was generated using the “addBgdPeaks” function. ChromVAR was performed with the “addDeviationsMatrix” function based on the CIS-BP motif database of 884 TFs to calculate deviation scores and variability for each TF motif sequence in every CD8^+^ T cells. The Z-score normalized TF motif deviation scores were imputed by MAGIC to visualize TF activity in every CD8^+^ T cell (Figs. 2B and S2B). Transcription factor footprinting was performed and visualized using the ArchR functions “getFootprints” and “plotFootprints” (flank = 200, normMethod = ‘subtract’, smoothWindow = 5) in Fig. S2C.

### TF motif deviation coexpression network analysis

To infer the crucial TF regulatory interactions, we used the TF motif deviation scores to construct the coexpression network for each sample. GENIE3 (Aibar et al., 2017) algorithm was used to infer gene regulatory networks based on the selected important TFs for each sample. The output coexpression network contained the input TFs, the inferred potential regulators, and their importance measure scores. Last, we used the importance measure scores as interaction weights to construct a directed TF regulatory network using the R package “igraph” (v1.2.5) in Fig. 2C.

### Gene signature enrichment in scATAC-seq data

Published CD8^+^ T-cell gene signatures were used to calculate the average expression of the genes in the signature list in every single cell using the “addModuleScore” function from ArchR. The gene signature scores were then imputed by MAGIC for visualization in every cell (Fig. 1E), and the box plot was plotted using the R package “ggplot2” (v3.3.5) in Fig. S1F. For effector CD8^+^ T cells, we also performed gene signature enrichment using the “addModuleScore” function to detect the characteristics of heterogeneity between SLEC and MPEC subsets and visualized in the UMAP embedding space in Fig. 4D.

### Reclustering analysis of effector and memory CD8^+^ T cells

To systematically dissect the epigenetic heterogeneity of effector and memory CD8^+^ T cells, we extracted the effector cells in D8_MPEC_^hi^ and D8 samples and memory cells in D180 sample for reclustering analysis, respectively. For effector cells, the selected subpopulations of cells from D8_MPEC_^hi^ and D8 samples were applied to cell clustering using the “FindNeighbors” (reduction=‘umap’, dims = 1:2) and “FindClusters” (algorithm = 1, resolution = 0.065) functions from Seurat based on the UMAP reduction and obtained four different clusters in Fig. 4A. For memory cells, we also conducted cell clustering with the pipeline as described above, with a lower resolution of 0.045 for “FindClusters” function (Fig. 5A). Next, to annotate the characteristics of different cell clusters in effector and memory CD8^+^ T cells, we checked the gene activity of well-defined marker genes of different T-cell subsets and assigned them as the known cell types. Peak calling, DARs identification, TF motif enrichment, and ChromVAR motif deviations were also conducted, as described above.

### Functional enrichment of differentially expressed genes (DEGs)

Differential gene activity was identified using the “getMarkerFeatures” and “getMarkers” functions for pairwise comparisons with “useMatrix = ‘GeneScoreMatrix’, bias = c(‘TSSEnrichment’, ‘log10[nFrags]’), testMethod = ‘Wilcoxon’, cutOff = ‘FDR <= 0.05 & Log2FC >= 0.5”) parameter settings. The gene ontology (GO) functional enrichment of DEGs was conducted using the Metascape web tool (Zhou et al., 2019). The pathways used for the enrichment analysis were obtained from the GO Biological Processes database (Ashburner et al., 2000; Gene Ontology Consortium, 2021). We obtained the DEGs between SLEC and MPEC subsets and conducted GO enrichment analysis in Fig. 4F and 4G. *P* values were calculated based on the accumulative hypergeometric distribution. Enriched GO terms with a *P* value < 0.01 were further selected and visualized in the Hiplot website (Li et al., 2022), a comprehensive web platform for scientific data visualization.

### Pseudotime trajectory inference analysis

To determine CD8^+^ T-cell differentiation trajectory based on progressive chromatin changes from naïve to effector and memory T-cell populations, pseudotime trajectory analysis was performed. Briefly, the slingshot (Street et al., 2018) (v1.8.0) R package was first used to infer the global lineage structure with a cluster-based minimum spanning tree based on reduced dimensional UMAP embeddings, and obtained two inferred trajectory (“Naïve->D2->D8_MPEC_^hi^->D8” and “Naïve->D2->D8_MPEC_^hi^->D180”) in Fig. S3A. Next, the ArchR object was subjected to “addTrajectory” function using the slingshot inferred trajectory as a guide to perform supervised trajectory analysis. The dynamics of peak accessibility, TF motif deviation scores, and gene activity scores along the pseudotime trajectory were calculated and visualized in the heatmap using the “getTrajectory” and “plotTrajectoryHeatmap” functions (Fig. 3B, 3D, 3E, 3I, 3K, and 3L).

For effector CD8^+^ T-cell subsets, we performed pseudotime trajectory analysis as described above. Accordingly, the slingshot was used to infer the trajectory and then imported to the ArchR object as a guide (“EEC1->SLEC” and “EEC1->EEC2->MPEC”) for supervised trajectory analysis using the “addTrajectory” function. For memory CD8^+^ T-cell subsets, pseudotime trajectory was also calculated based on the “TEM->TCM” differentiation using the “addTrajectory” function. The pseudotime trajectory heatmaps of peak accessibility, TF motif deviation scores, and gene activity scores were visualized using the “plotTrajectoryHeatmap” function (Figs. 4I, 4J, 4M, 4N, 5F, 5H, and 5I).

### OT-1 CD8^+^ T-cell single-cell ATAC-seq data analysis

The single-cell ATAC-seq data from OT-1 CD8^+^ T cells was also preprocessed and quantified using the 10x Genomics Cell Ranger ATAC pipeline (v1.2.0), and mapped to the mouse reference genome (mm10) as described above. Quality assessment and cell filtering for each sample were performed using the Signac (Stuart et al., 2021) (v1.1.1) package with following parameter settings: “peak_region_fragments > 2,000 & < 10,000, pct_reads_in_peaks > 40, blacklist_ratio < 0.02, nucleosome_signal < 10, TSS.enrichment > 2”. Next, to mitigate biological batch effects observed in different samples, we also utilized scAND (Dong and Zhang, 2021) to integrate multiple scATAC-seq datasets into a unified and unbatched dataset with parameter settings as mentioned above. Last, the scanpy (Wolf et al., 2018) (v1.6.0) package was applied to compute the neighborhood graph and dimensionality reduction using the “sc.pp.neighbors” and “sc.tl.umap” functions.

Following quality control and batch effect correction, the preprocessed scATAC-seq data was subjected to downstream analysis using the ArchR (Granja et al., 2021) (v1.0.1) R package with default parameters unless otherwise noted. Likewise, additional cell filtering was also conducted using ArchR for keeping only cell barcodes with at least 1000 fragments per cell and a TSS enrichment score ≥4. In total, we obtained 35,177 high-quality OT-1 CD8^+^ T cells from Naïve (11,165, 31.74%), D2 (11,594, 32.96%), and D8 (12,418, 35.30%) samples for subsequent analysis using the ArchR package as descripted above. Briefly, to reduce dimensionality, we performed LSI using the “addIterativeLSI” function with the following parameter settings: “useMatrix = ‘TileMatrix’, iterations = 4, varFeatures = 15,000, and dimsToUse = 1:30”. From the dimensionality reduction, we selected the top 30 dimensions for downstream analyses. Next, the precalculated UMAP dimensional embeddings computed by scanpy were incorporated into the ArchR object to visualize the dataset. Last, cell clustering was conducted using the “addClusters” function with “reducedDims = ‘IterativeLSI’, method = ‘Seurat’, resolution = 0.3” parameter settings, and four cell clusters were obtained and annotated according to the gene activity of cell type specific markers. Furthermore, gene activity calculation, peak calling and differential analysis, TF motif deviation enrichment, and pseudotime trajectory inference were also carried out in OT-1 CD8^+^ T cells as mentioned above. To compare the epigenetic similarities, we conducted principal component analysis (PCA) between P14 and OT-1 CD8^+^ T cells based on the gene activity of all genes calculated for each sample.

### RNA Pol II *in situ* ChIA-PET data processing

The data from mouse CD8^+^ T cell RNA Pol II were processed using a customized *in situ* ChIA-PET data processing pipeline, as described previously (Tang et al., 2015). Briefly, the bridge linker sequences were first detected in each sequenced PET read, and only the PETs with bridge linkers were kept for downstream analysis. Following the trimming of the bridge linkers, the sequences flanking the linker were mapped to the mouse reference genome mm10 using the “bwa-mem” command of the BWA (Li and Durbin, 2009) software (v0.7.9a) with default parameter settings. Only the uniquely mapped PETs with a mapping quality greater than 30 were retained using the Samtools (Li et al., 2009) software (v1.3.1). PCR duplicates were then removed using the “MarkDuplicates” function of the Picard Tools. The mapped reads statistics for each sample are summarized in Fig. S6A.

Each uniquely nonredundant PETs were then categorized as either a self-ligation PET (two ends of the same DNA fragment) or inter-ligation PET (two ends from two different DNA fragments in the same chromatin complex), depending on the genomic span between the two ends of a PET or origin of the two ends of a PET from two different chromosomes. PETs with both ends originating from the same chromosome and a genomic span of fewer than 8 kb were classified as self-ligation PETs. Self-ligation PETs were used as a proxy for ChIP fragments since they were derived in a manner analogous to ChIP-seq mapping for protein binding sites. PETs with both ends originating from the same chromosome and a genomic span greater than 8 kb were classified as inter-ligation PETs. PETs with each end from a different chromosome were also classified as intra-ligation PETs. The inter-ligation PETs reflect the long-range chromatin interaction mediated by proteins of interest.

To accurately represent the frequency of interaction between two loci, both ends of inter-ligation PETs were extended by 500 bp along the reference genome, and PETs overlapping at both ends (with extension) were grouped as a single PET cluster. Individual inter-ligation PETs that could not be merged as PET clusters were called singletons. A singleton is similar to Hi-C data in a function to reflect high-order chromatin topology (Rao et al., 2014; Tang et al., 2015). All uniquely mapped and nonredundant reads, including self-ligation and inter-ligation PETs, were used to calculate the RNA Pol II binding coverage along the chromosomes for visualization.

To obtain high-quality chromatin interaction regions mediated by RNAPII, we further filtered the PET clusters as follows. First, clusters with PET counts less than four were removed. Secondly, histone modifications of H3K27ac had to appear on both anchors of RNAPII. Finally, only clusters with a genomic distance of less than 1 Mb between the two anchors of the cluster were retained for visualization using the BASIC browser (Lee et al., 2020). These conserved clusters were RNAPII-mediated chromatin loops. We used MACS 1.4.2 (Zhang et al., 2008) with default parameters to identify binding peaks for the RNA Pol II coverage reads. The peak enrichment heatmap of each sample was calculated and visualized by the “computeMatrix” and “plotHeatmap” functions using the deepTools (Ramirez et al., 2016). The interaction contact heatmap was normalized by the HiCDCPlus (Sahin et al., 2021) R package and then visualized by the Juicebox (Robinson et al., 2018) and HiCExplorer (Wolff et al., 2018) python package in Fig. 6C–E.

### sh*Sox4* and vector transduced MPECs RNA-seq data analysis

For RNA-seq data analysis, the mouse reference genome (GRCm38/mm10) was first indexed using the Hisat2 (v2.1.0) (Kim et al., 2019) software for the alignment of paired-end RNA-seq reads. Clean reads were mapped to the mouse reference genome using Hisat2 (v2.1.0) with default parameters. HTSeq (v0.11.2) was employed to quantify the read counts for each gene in the mm10 genome assembly. We utilized the StringTie (v1.3.5) (Kovaka et al., 2019) software to calculate the Fragments Per Kilobase of transcript per Million mapped reads (FPKM) values, providing a robust quantification of gene expression levels across each sample. To visualize the gene expression profiles, we employed the pheatmap (v1.0.12) R package to create heatmaps that effectively display the expression patterns of representative genes under different conditions.

### Quantification and statistical analysis

Mice were randomly assigned to treatment groups and there were no inclusion/exclusion criteria. Statistical analysis was performed with Prism 7.0 software (GraphPad Software) by two-tailed paired Student’s *t*-test and two-tailed unpaired Student’s *t*-test. Graphs show individual samples and center values indicate mean. *P* values < 0.05 were considered significant (*: *P* < 0.05; **: *P* < 0.01; ***: *P* < 0.001 ; ****: *P* < 0.0001); ns: not significant, *P* values > 0.05.

## Supplementary Material

pwaf003_suppl_Supplementary_Material

pwaf003_suppl_Supplementary_Table_S1-S3

pwaf003_suppl_Supplementary_Table_S4

pwaf003_suppl_Supplementary_Table_S5-S8

## Data Availability

The raw data supporting the conclusions of this article will be made available by the authors, without undue reservation. The sequencing datasets generated in this study have been deposited at the Gene Expression Omnibus (GEO) under the accession numbers GSE226067, GSE241714, GSE226175, and GSE282282, which correspond to the scATAC-seq, ChIA-PET, and bulk RNA-seq datasets, respectively.

## Code availability

All data analyses were followed by standard programs and codes are available on reasonable request.
