# Supplementary material for: Chromatin landscape alteration uncovers multiple transcriptional circuits during memory CD8+ T-cell differentiation
Source: Protein Cell. 2025 Jan 13;16(7):575–601. doi: 10.1093/procel/pwaf003 (PMC12275094; doi:10.1093/procel/pwaf003)
Supplement: pwaf003_suppl_Supplementary_Material [file pwaf003_suppl_supplementary_material.docx]

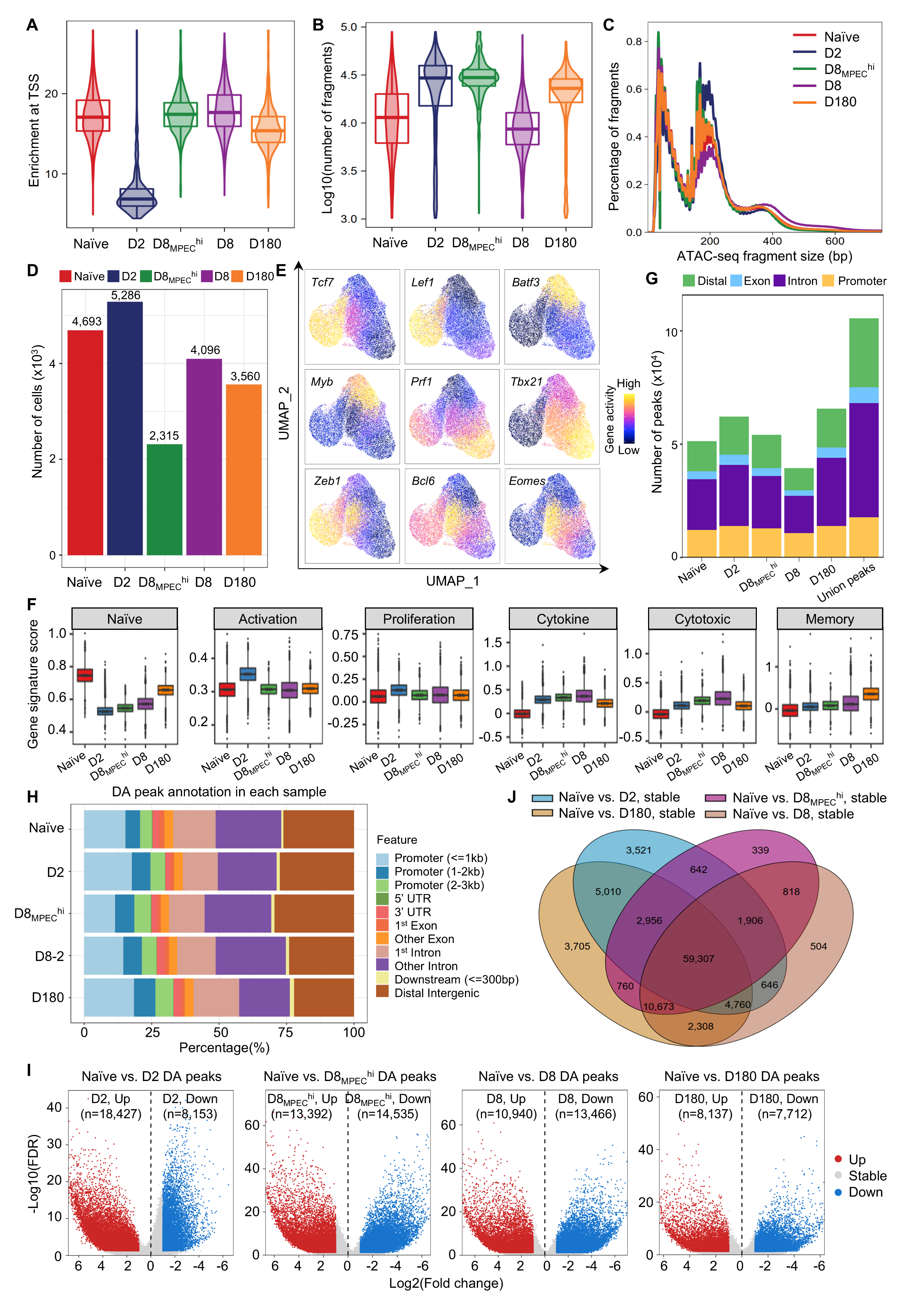


**Fig. S1. scATAC-seq revealed cell state-specific epigenetic features in response to acute viral infection, related to Fig. 1. (A)** Violin plot shows transcription start site (TSS) enrichment for all cells in each sample. The box plot denotes the medians and the quartile range (25-75%), and the length of whiskers represents 1.5 x the IQR. **(B)** The violin plot shows the number of fragments in log10 scale for all cells in each sample. **(C)** The density plot shows the ATAC-seq fragment size distribution in each sample. **(D)** The bar plot shows the number of cells in each sample. **(E)** UMAP visualization illustrates the activity scores of representative genes in all samples. The gene activity score was calculated by the aggregate accessibility of peaks linked to the associated genes. **(F)** The box plot shows the gene signature enrichment using the scATAC-seq dataset in each sample. **(G)** The stacked bar plot shows the peak number with genomic annotation in each sample. **(H)** The stacked bar plot shows the percentage of differentially accessible regions with genomic annotation for each sample. **(I)** The volcano plot displays the differential chromatin accessible peaks between naïve and other individual samples. **(J)** Venn diagram shows the number of stable peaks compared between naïve and other individual samples.


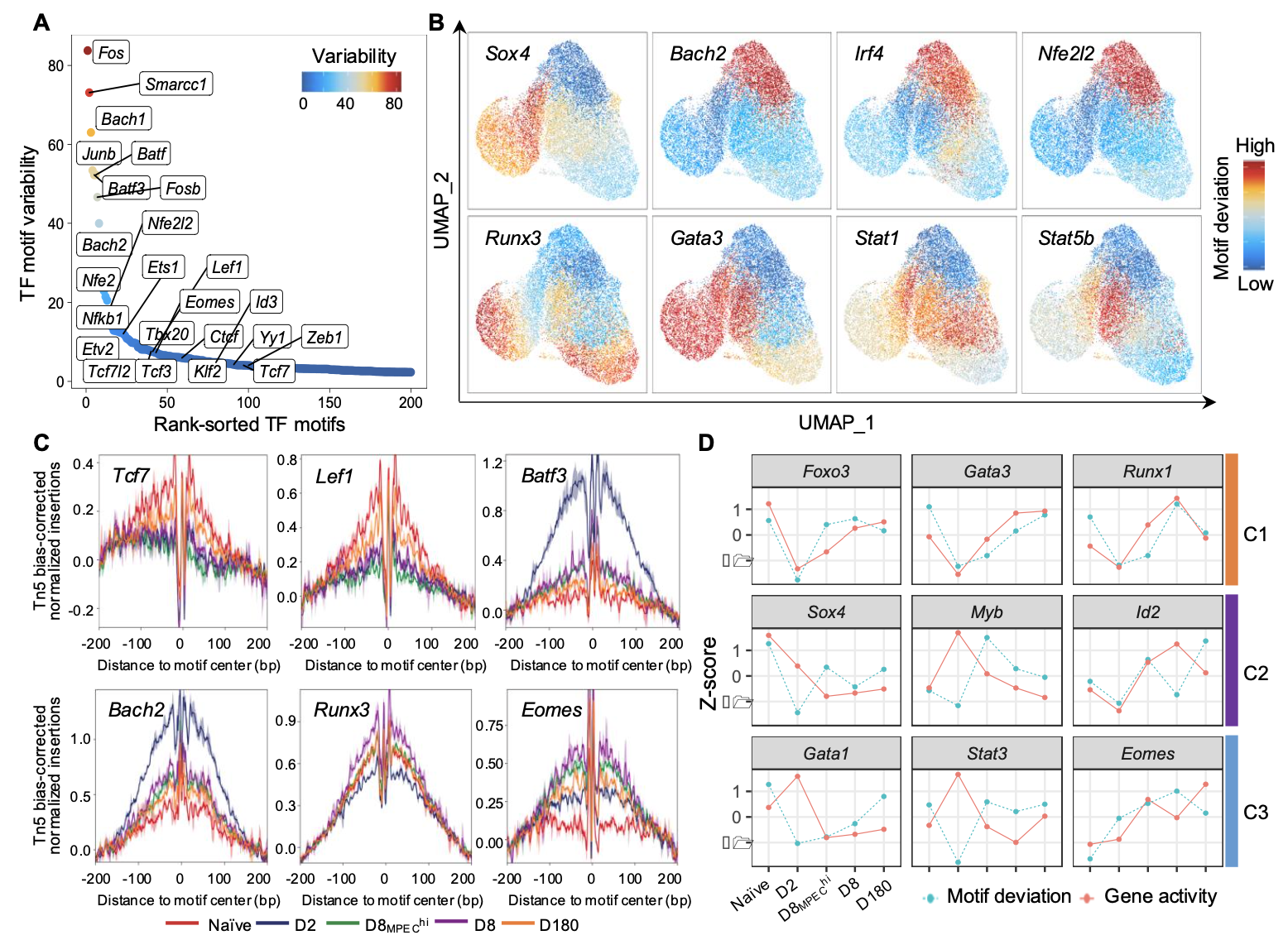


**Fig. S2. Distinct usage of transcriptional factors in CD8^+^ T-cells differentiation, related to Fig. 2. (A)** Ranking dot plot represents the motif variability detected by ChromVAR in all samples. The 200 top-ranked motifs were selected for the plot. **(B)** UMAP projection illustrates motif deviation scores in each cell. **(C)** The line plot shows transcription factor footprints of selected TF motifs using scATAC-seq data. **(D)** The line chart shows normalized z-scores of TF gene activity and the corresponding motif deviation across each sample.

**
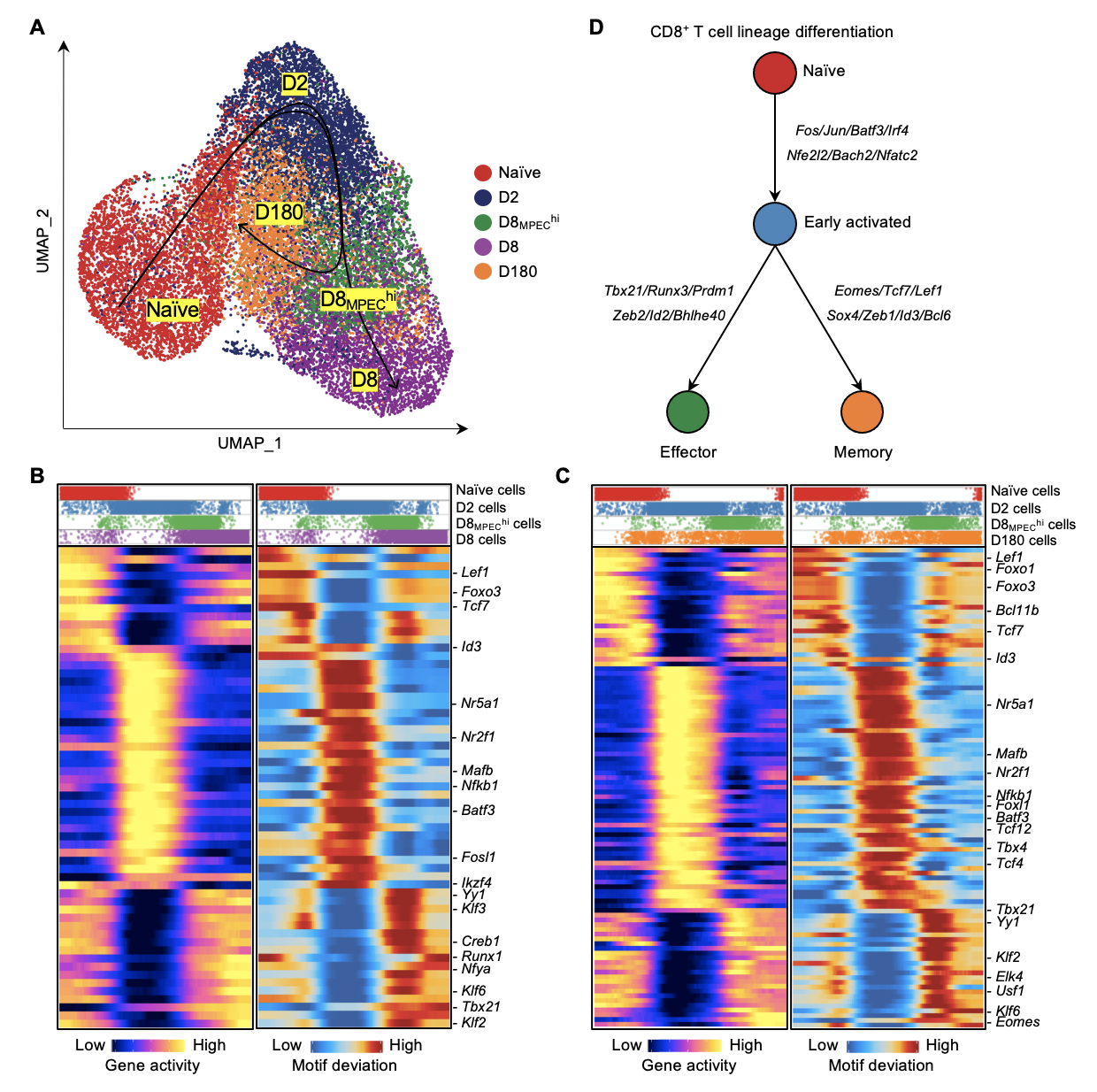
Fig. S3. Early activated CD8^+^ T cells undergo distinct epigenetic trajectories for developing effector and memory CD8^+^ T cells, related to Fig. 3. (A)** UMAP visualization shows the inferred trajectory for effector and memory CD8^+^ T cell differentiation. The smoothed line with the arrow represents the predicted trajectory path. **(B)** The heatmap shows the dynamic concordant TF gene activity (left) and motif deviation (right) along the pseudotime during effector CD8^+^ T-cell differentiation. Notable motifs/genes are indicated near their approximate positions in the heatmap. The top panel denotes the cell distribution along the pseudotime trajectory from each sample. **(C)** The heatmap shows the dynamic concordant TF gene activity (left) and motif deviation (right) along the pseudotime during memory CD8^+^ T-cell differentiation. Similar to **(B)**. **(D)** A schematic diagram shows the differential regulatory TFs driving CD8^+^ T-cell differentiation from naïve to effector or memory state.

**
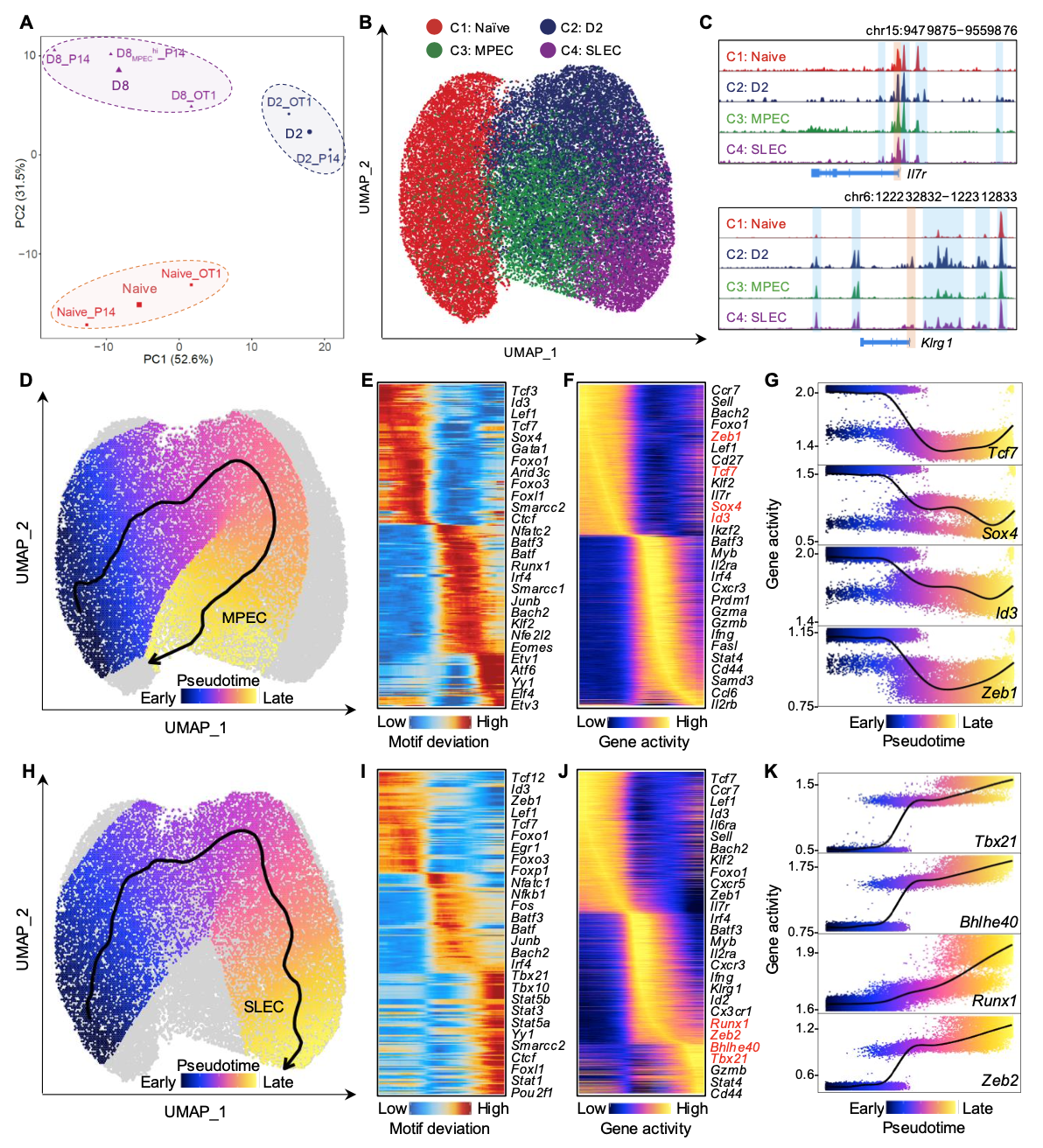
**

**Figure S4. OT-1 CD8^+^ T cells show similar epigenetic reprogramming in response to acute bacterial infection, related to Figure 3**. **(A)** Principal component analysis (PCA) plot of OT-1 and P14 CD8^+^ T cells from different acute infection models. **(B)** UMAP projection of four clustered 35,177 CD8^+^ T cells from three samples with single-cell chromatin accessibility profiles. **(C)** The genome track view visualizes the chromatin accessibility at *Il7r* and *Klrg1* loci by aggregating scATAC-seq signals from each cell cluster. Promoters and proximal cis-regulatory elements are highlighted in boxes. **(D)** UMAP visualization displays the inferred differentiation trajectory from Naïve to MPEC cells. UMAP embedding overlaid with pseudotime values of individual cells. The smoothed line with the arrow represents the predicted trajectory path. **(E, F)** Heatmap shows motif deviation **(E)** and gene activity **(F)** scores along pseudotime from naïve to MPEC differentiation trajectory. **(G)** Scatter plot shows ordered activity scores of representative genes over pseudotime of the differentiation trajectory from Naïve to MPEC cells. A smooth line represents loess regressions for gene activity scores. Each dot represents an individual cell. **(H)** UMAP visualization displays the inferred differentiation trajectory from naïve to SLEC cells. Similar to (D). **(I, J)** Heatmap shows motif deviation **(I)** and gene activity **(J)** scores along pseudotime from Naïve to SLEC differentiation trajectory. **(K)** Scatter plot shows ordered activity scores of representative genes over pseudotime of the differentiation trajectory from Naïve to SLEC cells. Similar to **(G)**.


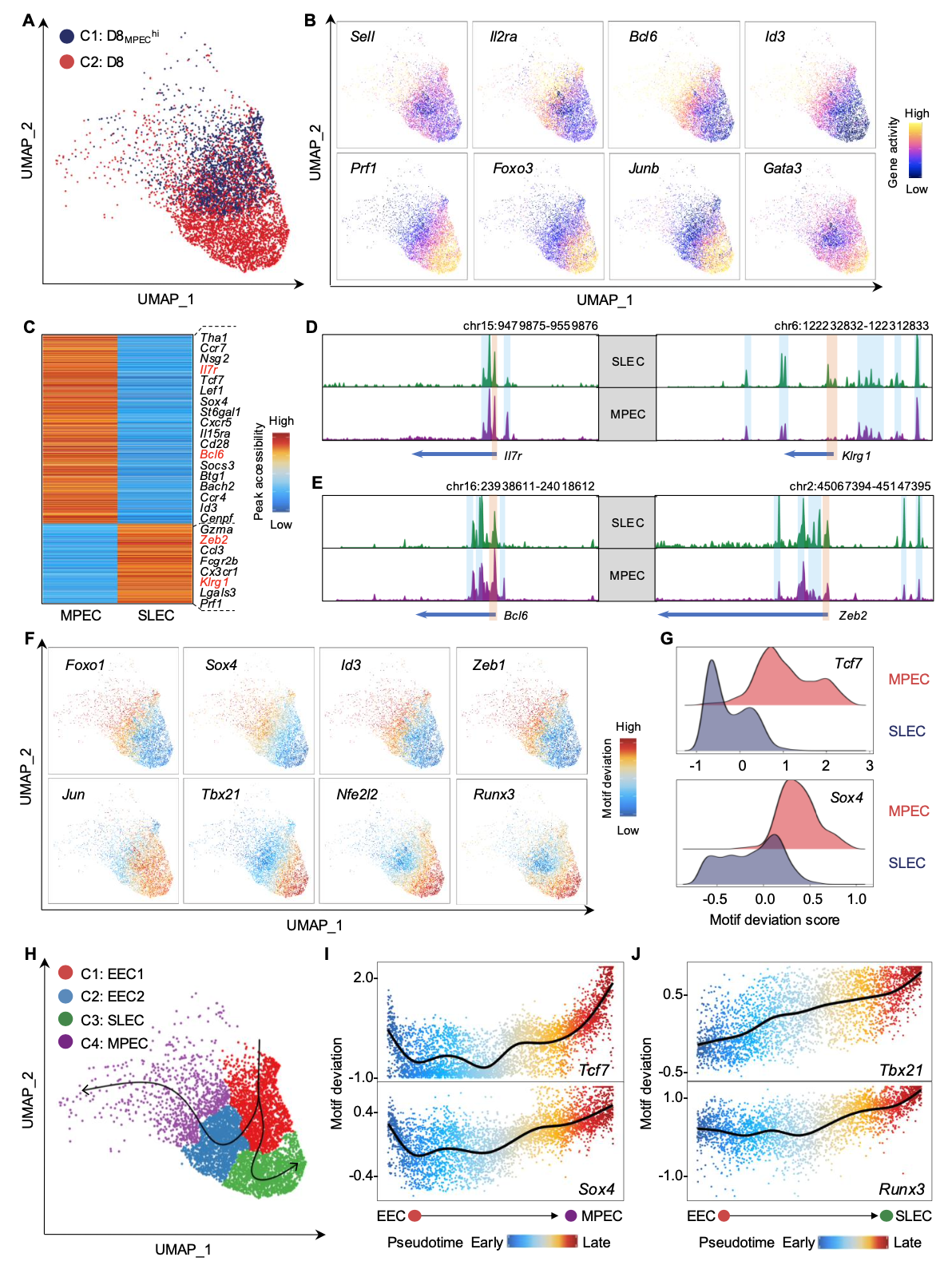


**Fig. S5. Effector CD8^+^ T cells are epigenetic heterogeneity, related to Fig. 4.** **(A)** UMAP visualization shows clustered effector CD8^+^ T cells from D8_MPEC_^hi^ and D8 samples. **(B)** UMAP visualization illustrates activity scores of representative genes for clusters in **(A)**. The gene activity score was calculated by the aggregate accessibility of peaks linked to the associated genes. **(C)** The heatmap shows normalized chromatin accessibility between MPEC and SLEC cells. Notable genes are indicated near their approximate positions in the heatmaps. **(D, E)** Genome track view visualizes the chromatin accessibility at *Il7r*, *Klrg1* **(D)** and *Bcl6*, *Zeb2* **(E)** locus by aggregating scATAC-seq signals from each cluster. Promoters and *cis*-regulatory elements are highlighted in boxes. **(F)** UMAP projection illustrates TF motif deviation scores in each cell. **(G)** The ridge plot shows *Tcf7* and *Sox4* motif deviation scores between MPEC and SLEC cells. **(H)** UMAP visualization displays the inferred differentiation trajectories for developing MPEC and SLEC cells. The smooth line with the arrow represents the predicted trajectory path. **(I)** Scatter plot shows ordered motif deviation scores of *Tcf7* and *Sox4* over pseudotime of the differentiation trajectory from EEC to MPEC cells. A smooth line represents loess regressions for motif deviation scores. **(J)** Scatter plot shows ordered motif deviation scores of *Tbx21* and *Runx3* over pseudotime of the differentiation trajectory from EEC to SLEC cells. Similar to **(I)**.


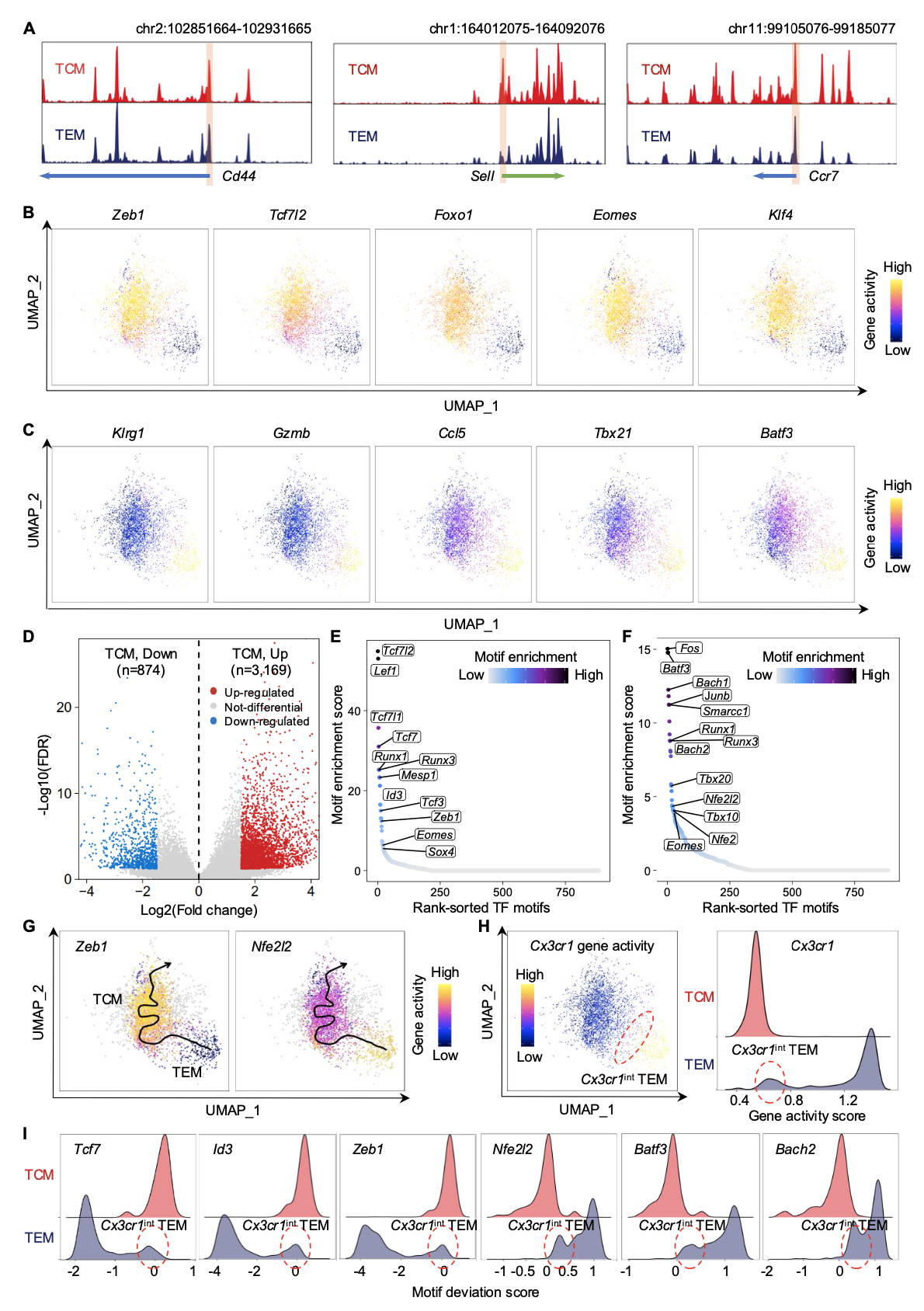
**Fig. S6. Memory T cells show epigenomic heterogeneity, related to Fig. 5.** **(A)** Genome track view visualizes the chromatin accessibility at *Cd44*, *Sell*, and *Ccr7* loci by aggregating scATAC-seq signals from TCM and TEM subsets. Promoters are highlighted in boxes. **(B, C)** UMAP visualization illustrates gene activity scores of representative highly accessible genes in TCM **(B)** and TEM **(C)** cells. The gene activity score was calculated by the accessibility of peaks linked to the associated genes. **(D)** The volcano plot demonstrates the differential chromatin accessible peaks between TCM and TEM cells. **(E, F)** Ranking dot plot shows the enrichment of TF motifs in TCM **(E)** and TEM **(F)** differentially accessible peaks. **(G)** UMAP plot shows *Zeb1* and *Nfe2l2* gene activity scores over pseudotime of the differentiation trajectory from TEM to TCM cells. The smoothed line with an arrow represents the predicted trajectory path. **(H)** UMAP (left) and ridge (right) plots show the gene activity score of *Cx3cr1* between TEM and TCM cells. **(I)** The ridge plot shows the TF motif deviation scores of *Tcf7*, *Id3*, *Zeb1*, *Nfe2l2*, *Batf3*, and *Bach2* between TEM and TCM cells.


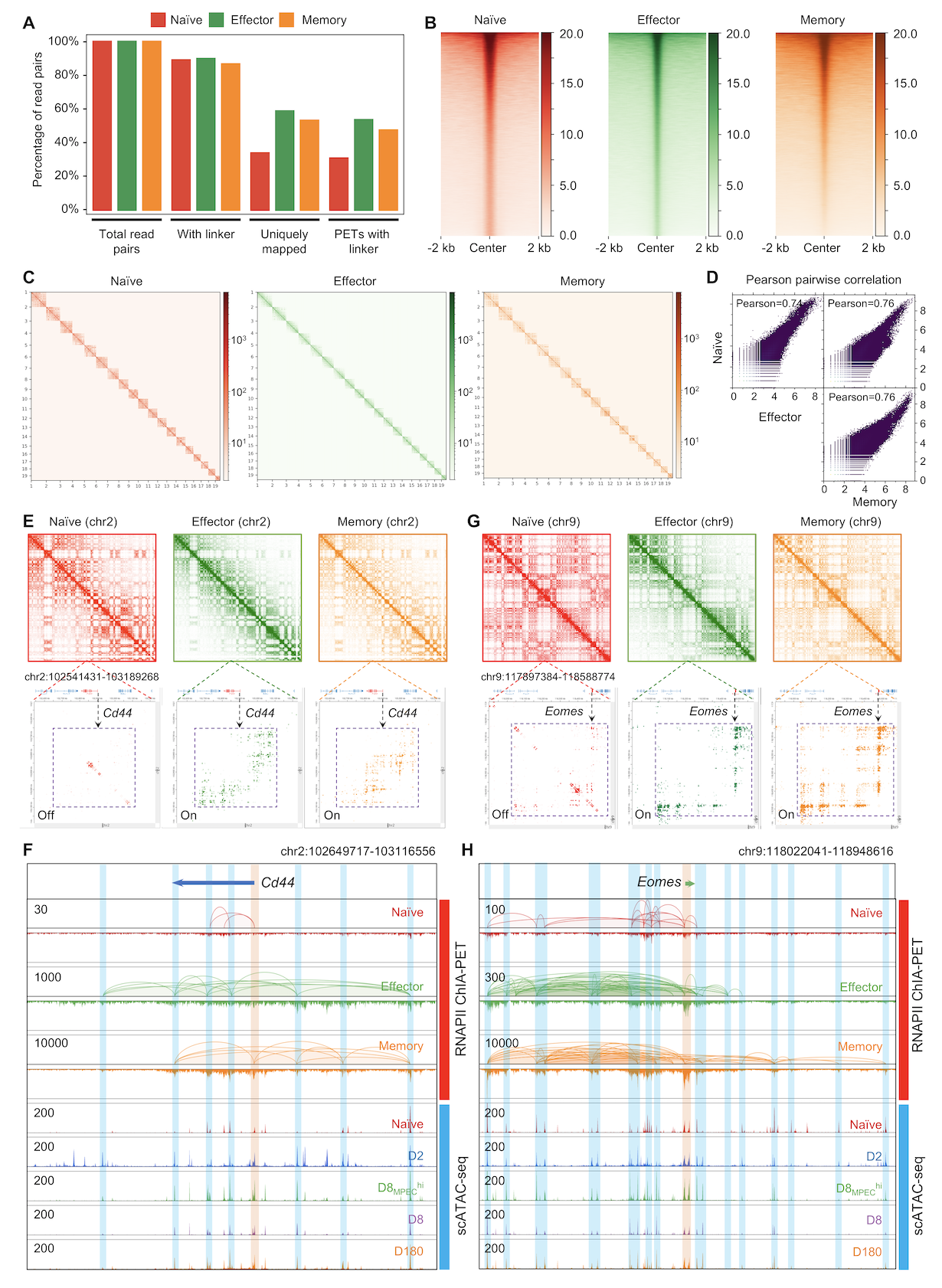


**Fig. S7. Dynamic changes in enhancer-promotor chromatin interactions during CD8^+^ T-cell differentiation, related to Fig. 6.** **(A)** Bar plot shows the percentage of categorized read-pairs in RNAPII ChIA-PET data for each sample. **(B)** The heat map shows the pileup intensity of reads aligned around peaks (each row) for each sample. **(C)** 2D contact map shows RNAPII-mediated intra-chromosomal chromatin interactions across all chromosomes for each sample. **(D)** Scatter plot shows the pairwise Pearson correlations between each of the two samples. **(E, G)** 2D contact map shows RNAPII-mediated chromatin interactions on chromosomes 2 **(E)** and 9 **(G)** (top) and zoomed-in regions of *Cd44* **(E)** and *Eomes* **(G)** loci (bottom) from naïve, effector, and memory CD8^+^ T cells. The ‘On’ and ‘Off’ labels in the left corner of bottom panels indicated the gene activities according to RNAPII looping intensity at *Cd44* and *Eomes* loci. **(F, H)** The genome browser view shows chromatin interaction loops and binding peaks detected by RNAPII ChIA-PET for naïve, effector, and memory CD8^+^ T cells and aggregated pseudo-bulk ATAC-seq signals of each sample at the *Cd44* **(F)** and *Eomes* **(H)** loci. The promoter and enhancer peaks are highlighted in boxes, respectively.


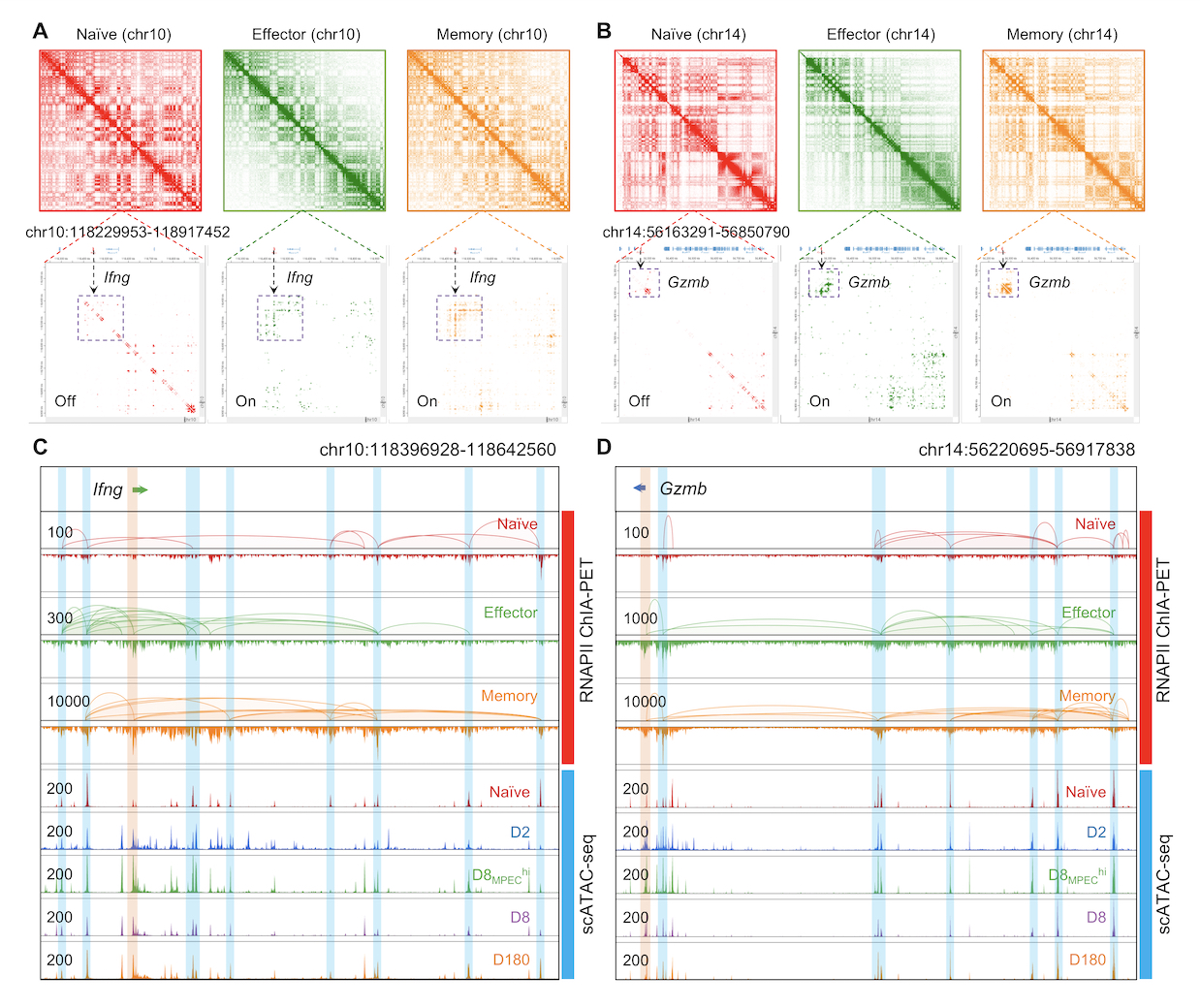


**Fig. S8.** **Dynamic enhancer-promotor chromatin interactions** **contribute to alterations in gene expressions, related to Fig. 6. (A, B)** 2D-contact map shows RNAPII-mediated chromatin interactions on chromosomes 10 **(A)** and 14 **(B)** (top) and zoomed-in regions of *Ifng* **(A)** and *Gzmb* **(B)** loci (bottom) from naïve, effector, and memory CD8^+^ T cells. The ‘On’ and ‘Off’ labels in the left corner of the bottom panels indicated the gene activities according to RNAPII looping intensity at *Ifng* and *Gzmb* loci. **(C, D)** The genome browser view shows chromatin interaction loops and binding peaks detected by RNAPII ChIA-PET for naïve, effector, and memory CD8^+^ T cells and aggregated pseudo-bulk ATAC-seq signals of each sample at the *Ifng* **(C)** and *Gzmb* **(D)** loci. The promoter and enhancer peaks are highlighted in boxes, respectively.

**
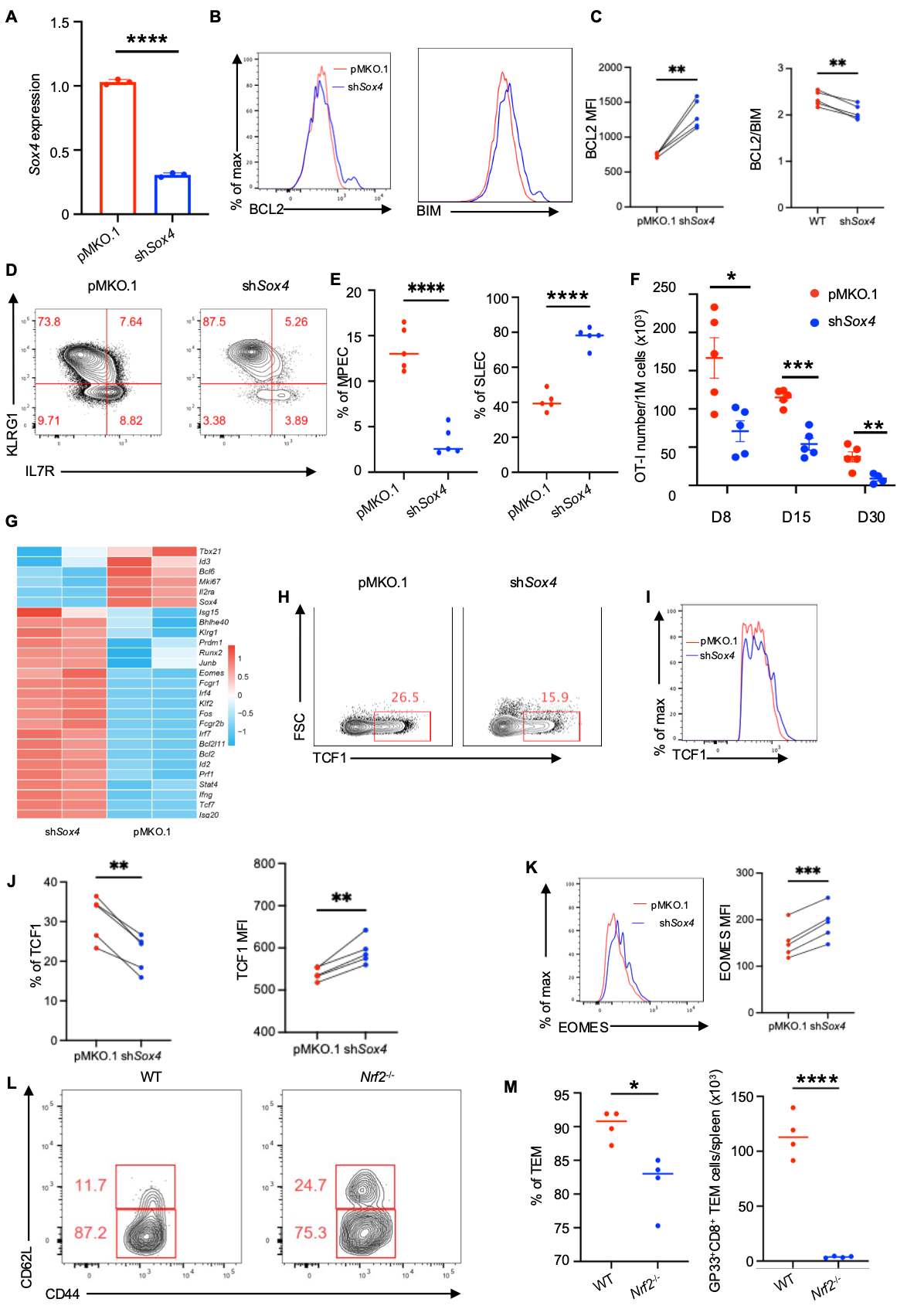
Fig. S9. *Sox4* and *Nrf2* are critical for memory T-cell differentiation, related to Fig. 7.**

**(A)** Box plot shows the expression level of *Sox4* between pMKO.1 and sh*Sox4* P14 MPEC cells. **(B)** FACS analyses of BCL2 and BIM expression in indicated MPECs of P14 cells. **(C)** The MFIs of BCL2 in each population (left). The ratio of BCL2/BIM in each population(right). **(D,E)** CD45.1^+^CD8^+^ OT-1 cells were transduced with retrovirus encoding short hairpin RNA (shRNA) targeting *Sox4* (sh*Sox4*) or vector (pMKO.1), the GFP^+^ cells were sorted and transferred into CD45.2^+^ recipient mice separately, followed by infection with Lm-OVA, the differentiation of CD8^+^ T cells were monitored. Representative flow cytometry plots from eight-days after Lm-OVA infection **(D)**. Enumeration of MPEC and SLEC subsets from Lm-OVA infection as gated in the left panel **(E)**. **(F)** The dot plot shows the absolute number of OT-1 cells among different time points between pMKO.1 and sh*Sox4* OT-1 cells in the blood, Data are presented as mean values ± SEM. **(G)** The heatmap shows the gene expression profiles of representative genes between sh*Sox4* and vector-transduced MPECs. **(H)** The representative flow cytometry plot shows the percentage of TCF1 positive cells from D8 LCMV-Arm^+^ infection. Cells were gated on Live vector or *shSox4* transduced P14 cells. **(I)** FACS analyses of TCF1 expression in indicated MPECs of P14 cells. **(J)**The percentage and MFIs (mean fluorescence intensity) of TCF1 in each MPECs population(right). **(K)** FACS analyses of EOMES expression in indicated MPECs of P14 cells (left). The MFIs of EOMES in each population (right). **(L)** Representative flow cytometry of TCM (CD44^+^CD62L^+^) and TEM (CD44^+^CD62L^-^) cells in wild-type and *Nrf2*^-/-^ mice, cells were gated on live GP33^+^CD8^+^ T cells. **(M)** The dot plot shows the percentage and absolute number of GP33^+^CD8^+^ TEM cells between wild-type and *Nrf2*^-/-^ mice.
